# Integrating network pharmacology and experimental validation to clarify the anti-hyperuricemia mechanism of cortex phellodendri in mice

**DOI:** 10.3389/fphar.2022.964593

**Published:** 2022-11-10

**Authors:** Lieqiang Xu, Juanjuan Cheng, Jieyi Lu, Guoshu Lin, Qiuxia Yu, Yucui Li, Jiannan Chen, Jianhui Xie, Ziren Su, Qi Zhou

**Affiliations:** ^1^ College of Bioscience and Bioengineering, Jiangxi Agricultural University, Nanchang, China; ^2^ School of Pharmaceutical Sciences, Guangzhou University of Chinese Medicine, Guangzhou, China; ^3^ The Second Affiliated Hospital of Guangzhou University of Chinese Medicine, Guangzhou, China; ^4^ China Resources Sanjiu Medical & Pharmaceutical Co., Ltd., Shenzhen, China

**Keywords:** Cortex Phellodendri, hyperuricemia, network pharmacology, berberine, xanthine oxidase

## Abstract

Hyperuricemia (HUA), a common metabolic disease, is treated as the second-largest metabolic disease after diabetes in China. Cortex Phellodendri (CP) is one of the most frequently used herbal medicines for treating gout or HUA. However, the mechanism underlying the anti-HUA effect of CP is still unrevealed. Hence, this study aimed to explore the pharmacological mechanism of CP against HUA using network pharmacology coupled with *in vivo* experimental validation. Active compounds and potential targets of CP, as well as the potential targets related to HUA, were retrieved from multiple open-source databases. The drug-disease overlapping targets were obtained by Venn diagram analysis and used to construct the herb-component-target (HCT), protein-protein-interaction (PPI), and component-target-pathway (CTP) networks. The functional enrichment analysis was also performed for further study. Furthermore, a HUA mouse model was induced by a combination of intraperitoneal injection of potassium oxonate (PO, 300 mg/kg) and intragastric administration of hypoxanthine (HX, 300 mg/kg) daily for 10 days. Different dosages of CP (200, 400, and 800 mg/kg) were orally given to mice 1 h after modeling. The results showed that 12 bioactive compounds and 122 drug-disease overlapping targets were obtained by matching 415 CP-related targets and 679 HUA-related targets, and berberine was one of the most important compounds with the highest degree value. The core targets of CP for treating HUA were TP53, MAPK8, MAPK3, IL-6, c-Jun, AKT1, xanthine oxidase (XOD), and ATP-binding cassette subfamily G member 2 (ABCG2). The Kyoto Encyclopedia of Genes and Genomes (KEGG) enrichment results showed that the anti-HUA effect of CP mainly involved the pathways of inflammation and apoptosis, such as PI3K/Akt, TNF, MAPK, TLR, AMPK, NF-κB, and NLRP3 signaling pathways. *In vivo* animal experiment further confirmed the hypouricemic effect of CP in a HUA mouse model, as evidenced by significantly restored kidney histological deteriorations, and considerably decreased levels of serum uric acid (sUA), creatinine (Cre), blood urea nitrogen (BUN), and hepatic UA. Furthermore, the hypouricemic action of CP *in vivo* might be attributed to its suppression of XOD activity in the liver, rather than ABCG2 in the kidney. Real-time qPCR (RT-qPCR) and Western blot analysis also confirmed the key roles of the hub genes in CP against HUA. In conclusion, CP exhibited therapeutic effect against HUA via multi-compounds, multi-targets, and multi-pathways. It possessed anti-HUA and nephroprotective effects via suppressing XOD activity, and reversed the progression of renal injury by exerting anti-inflammatory and anti-apoptotic effects.

## Introduction

Uric acid (UA) is the major final oxidation product of purine catabolism in humans and is formed by the catalytic action of xanthine oxidase (XOD). XOD is a key enzyme in the liver that catalyzes the transformation of hypoxanthine to xanthine and then to UA (Zhang et al., 2018). The presence of an increased UA level may be due to excessive production or decreased excretion, which is a sign of hyperuricemia (HUA). The cut-off value of 360 μmol/L for women and 420 μmol/L for men has been proposed to define HUA, considering the risk of monosodium urate crystal (MSU) formation increases when the sUA level exceeds the saturation threshold (Bardin and Richette, 2014). Excessive MSU deposits in the joints and soft tissues, triggering gout, tophi formation, kidney stones, and acute kidney failure (Richette and Bardin, 2010). Moreover, HUA is also closely related to the development of diabetes, kidney disease, hypertension, cardiovascular disease, and other diseases (Richette and Bardin, 2010). Urate-lowering therapy (ULT) has been widely adopted in the prevention and treatment of HUA and gout by reducing UA levels. However, most of the ULT agents in clinical practice have severe side effects or are ineffective for some patients (Pillinger and Mandell, 2020). Therefore, the development of effective and safe anti-HUA drugs remains an unmet clinical need.

Cortex Phellodendri (CP), also called “Huangbo” in Chinese, is a well-known Chinese medicine with two official monographs listed in the Chinese Pharmacopoeia: Cortex Phellodendron chinensis (CPC) and Cortex Phellodendron amurensis (CPA) (Commission, C. P., 2020). CPC (known as “Chuanhuangbo” in Chinese) is derived from the dried bark of *Phellodendron chinense* Schneid. and is mainly distributed in the southwest part of China. CPA (known as “Guanhuangbo” in Chinese) is derived from the dried bark of *Phellodendron amurense* Rupr. and is mainly distributed in the northeastern part of China. Generally, these two species are used interchangeably for the treatment of dysentery, jaundice, urinary infection, and skin eczema, especially HUA (Chen et al., 2010). CP is a core and fundamental herb utilized either alone or in combination with other herbs to treat HUA. Ermiao wan (EMW), a famous traditional Chinese medicinal (TCM) herbal prescription containing Atractylodis Rhizome and Cortex Phellodendri at a rate of 1:1 (w/w), was originally recorded in “Dan Xi Xin Fa” by Zhu Danxi in the Yuan Dynasty. EMW has been used medicinally for centuries for the treatment of gout and HUA, and modern pharmacological studies have also confirmed the anti-HUA effect of EMW (Kong et al., 2004; Wei et al., 2018). Due to its excellent hypouricemic ability, Sanmiao wan (Wang et al., 2010), Simiao wan (Ma et al., 2015), Jiawei Simiao wan (Hua et al., 2012), and Tongfeng decoction (Wu et al., 2019) were all invented based on EMW and exhibited potent anti-HUA and anti-gout effects. CP is the core herb among these herbal formulas, however, only a few studies have reported its urate-lowing effect (Yang et al., 2005; Pan et al., 2008). And the detailed mechanism underlying the hypouricemic and nephroprotective effects of CP remains obscure.

**FIGURE 1:**
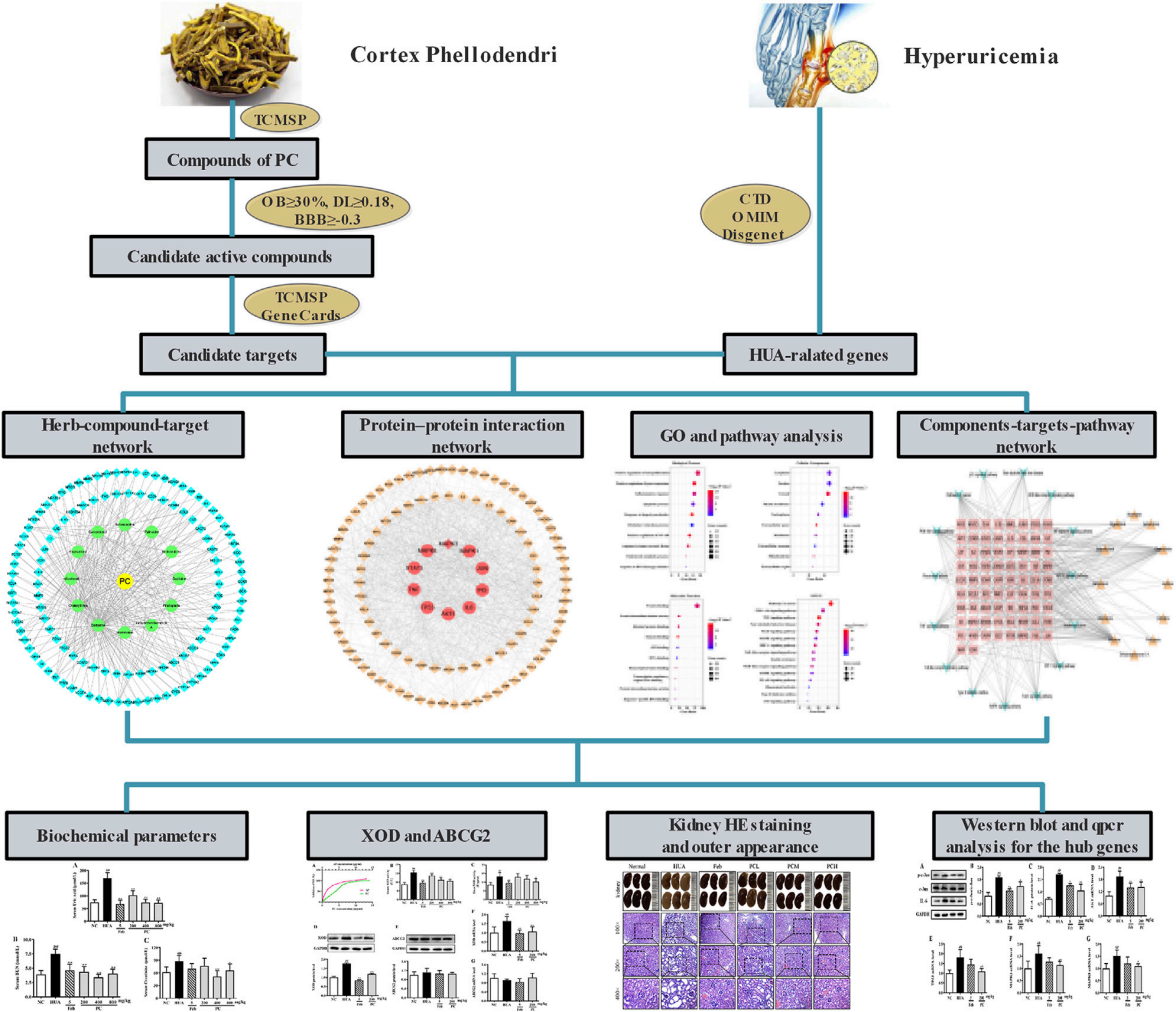
The flowchart of Phellodendri Chinensis Cortex for the treatment of HUA. TCMSP: Traditional Chinese Medicine Systems Pharmacology database; OB: oral bioavailability; DL: drug-like quality; BBB: blood-brain barrier; CTD: Comparative Toxicogenomics Database; OMIM; Online Mendelian Inheritance in Man.

TCM emphasizes a holistic and systematic theory of the occurrence and development of disease. TCM possesses advantages of multi-components and being applied with multiple targets as well as multi-pathways synergies in the treatment and prevention of diseases (Wang et al., 2020). The emerging network pharmacology is a reliable and efficient approach for TCM pharmacological research. And it has been widely applied to predict the complex mechanisms of TCM from a comprehensive perspective, which is in line with the holistic view, systematic approach, and compatibility principle of TCM (Berger and Iyengar, 2009; Dong et al., 2021). In this work, a network pharmacology approach was adopted to analyze the active ingredients, specific targets, and underlying molecular mechanisms of CP against HUA. Furthermore, a HUA mouse model was also constructed to assess the therapeutic effect of CP and to verify the potential molecular mechanism predicted by network pharmacology. These results not only shed light on the molecular mechanisms of CP for treating HUA, but also provided scientific evidence to support the expansion of its use in clinical applications. The workflow is shown in Figure 1.

## Materials and methods

### Reagents and materials

Potassium oxonate (PO, purity >97%, Cat#: 156124) and hypoxanthine (HX, purity >99%, Cat#: H9377) were bought from Sigma-Aldrich Chemical Co. (St. Louis, MO, United States). Febuxostat (Feb, purity >98%, Cat#: S42697) was purchased from Shanghai Yuanye Bio-Technology Co., Ltd. (Shanghai, China). Allopurinol (AP, purity >98%, Cat#: 204337) was purchased from Hebei Bailing Weichao Fine Materials Co., Ltd. (Langfang, China). UA assay kits that used the enzymatic-colorimetric method (Cat#: C012-2-1), Cre assay kits that used the sarcosine oxidase method (Cat#: C011-2-1), and BUN assay kits that used the urease method (Cat#: C013-2-1) were purchased from Nanjing Jiancheng Bioengineering Institute (Nanjing, China). The BCA protein determination kit (Cat#: P0012) was purchased from Beyotime Biotechnology (Shanghai, China). The following primary antibodies: rabbit anti-c-Jun pAb (Cat#: 24909-1-AP), rabbit anti-p-c-Jun pAb (Cat#: 28891-1-AP), rabbit anti-ABCG2 pAb (Cat#: 27286-1-AP), and anti-GAPDH pAb (Cat#: 10494-1-AP) were obtained from Proteintech Group, Inc. (Wuhan, China). Rabbit anti-XOD mAb (Cat#: ab109235), rabbit anti-IL-6 mAb (Cat#: ab233706), and the second antibody anti-rabbit IgG (Cat#: ab6721) were purchased from Abcam (Cambridge, MA, United States). All other reagents were at least of analytical grade.

### Preparation of ethanol extract

CPC was purchased from the First Affiliated Hospital of Guangzhou University of Chinese Medicine (GZUCM), Guangzhou, China. The herbal materials were extracted according to our previously published method (Xian et al., 2013). Briefly, the dried herbal material was crushed into powder by a pulverizer, passed through a 40-mesh screen, and 100 g of powder was refluxed with 800 ml of 80% aqueous ethanol for 1 h. The extraction procedure was then repeated twice more. The extract was pooled, filtered, and concentrated under reduced pressure in a rotary evaporator, followed by freeze-drying. The crude ethanol extract was resuspended in 0.5% sodium carboxymethyl cellulose (CMC-Na) for subsequent intragastric administration. The components of CPC extract have been detected and analyzed using high performance liquid chromatography, and the results have been shown in previous publication (Xian et al., 2013).

### Screening potential bioactive compounds and targets

The main active compounds of CP were collected from the TCMSP database (https://old.tcmsp-e.com/tcmsp.php) (Ru et al., 2014), the TCM Database@Taiwan (http://tcm.cmu.edu.tw) (Chen, 2011), and the TCMID database (http://www.megabionet.org/tcmid/) (Huang et al., 2018). According to the general filtering criteria suggested by TCMSP, the active compounds with OB ≥ 30%, DL ≥ 0.18 and BBB ≥ -0.3 were screened out for further research. Then the potential targets of the active substances of CP were identified by the TCMSP, SwissTargetPrediction (http://www.swisstargetprediction.ch/) (Daina et al., 2019) and Gene Cards databases (http://www.genecards.org/) (Safran et al., 2002). All targets were restricted to human origins. All filtered targets were further normalized to official gene symbols using the UniProt database (http://www.uniprot.org) (UniProt Consortium, 2019). Genes with missing annotations and duplicate genes were removed.

#### Screening potential disease targets of HUA

Here we applied the keyword of hyperuricemia to retrieve the related targets of disease in the following databases: CTD (http://ctdbase.org/) (Davis et al., 2021), OMIM (https://omim.org/) (Amberger et al., 2015), and DisGeNET (https://www.disgenet.org/) (Piñero et al., 2020). The overlapping targets between HUA and active compounds were obtained and visualized by the Venny (https://bioinfogp.cnb.csic.es/tools/venny/) online tools. The overlapping target set was considered the potential target of CP associated with HUA.

#### GO and KEGG pathway enrichment analysis

Gene Ontology (GO) terms and Kyoto Encyclopaedia of Genes and Genomes (KEGG) pathway enrichment analyses were assessed using the DAVID Bioinformatics Resources 6.8 (https://david.ncifcrf.gov/). The results were visualized as bubble charts using the RStudio software.

#### Network construction

To comprehensively elucidate the molecular mechanisms of CP in the treatment of HUA, the herb-component-target (HCT), protein-protein-interaction (PPI), and component-target-pathway (CTP) networks were constructed. Initially, to identify the core ingredients of CP treatment, the HCT network was established based on the active ingredients and overlapping genes. Furthermore, these overlapping genes were imported into the String database version 11.0 (https://string-db.org/) with Homo sapiens as the organism and a high confidence interaction score of >0.7. The interaction results were exported as a “TSV” format and imported into Cytoscape software 3.7.1 for building a PPI network and network analysis. Finally, on the basis of the HCT network and KEGG pathway analyses, the effective components, targets, and top 15 pathway information were imported to Cytoscape software to construct the CTP network. The topological structure of networks was analyzed using the Network Analyzer and the CytoHubba plugin. The core ingredients and genes were ranked by degree value.

#### Animals

Six-to eight-week-old male ICR mice, weighing 18–22 g, were obtained from the Laboratory Animal Center of GZUCM. Before performing any experimental procedure, all mice were supplemented with a regular diet and allowed to drink *ad libitum* for 7 days to adapt to the controlled environment (22 ± 2°C, 60 ± 10% relative humidity, fixed 12-h artificial photoperiod). The animal experimental procedures were approved by the Animal Ethics Committee of GZUCM.

After acclimatization for 1 week, 72 male animals were assigned to 6 groups (*n* = 12/group): normal control (NC), HUA, febuxostat (5 mg/kg), and CP group (200, 400, and 800 mg/kg). To establish a hyperuricemic mouse model, all animals except normal mice were administrated with 300 mg/kg HX orally and 300 mg/kg PO intraperitoneally for 10 days. Mice in NC group mice were administered orally with an equal amount of normal saline (NS, 0.9%). One hour after modeling, animals in the treatment groups were orally given three doses (200, 400, and 800 mg/kg) of CP, while mice in the NC and HUA groups were given equal amounts of NS instead. Febuxostat was given as a positive control. All animals were anesthetized with pentobarbital sodium (60 mg/kg) 4 h after CP treatment on day 10. All efforts were made to minimize suffering. The blood samples were collected by retro-orbital bleeding for serum preparation. Tissues were rapidly dissected out, rinsed, blotted dry, weighed, frozen in liquid nitrogen, and then stored at −80°C until processed.

#### Biochemical assays

The whole blood was centrifuged at 3500 rpm for 10 min at 4°C immediately after blood collection. The supernatant containing the serum was collected and then analyzed immediately for levels of UA, BUN, Cre, and XOD activity using appropriate commercial kits according to the manufacturer’s protocols.

The liver tissues were homogenized in NS for 3 min at a ratio of 10% w/v using a homogenizer. The homogenate was centrifuged at 3,000 rpm at 4°C for 15 min. The total protein concentration was measured with a BCA assay kit. The supernatant fraction was used to measure the UA level and XOD activity in the liver using appropriate commercial kits according to the manufacturer’s protocols.

#### Histological assays

The kidney tissues were fixed in 4% paraformaldehyde for 24 h at room temperature, and then routinely processed for paraffin embedding, sectioned at 5 μm, and stained with hematoxylin-eosin (HE). Then the stained kidney slices were imaged under Olympus BX53 optical microscopy (Tokyo, Japan) at 100 ×, 200 ×, and 400 × magnification. Additionally, histopathological scores were assessed double-blindly according to the methods established by a previous study (Xu Y. et al., 2021). Generally, the method was based on glomerulopathy (0–4, from normal glomerular structure to most glomerular atrophy); tubulopathy (0–4, from normal structure of tubules to most tubule dilation); and renal interstitial inflammatory infiltration (0–4, from non-inflammatory cells to a large number of inflammatory cells).

#### XOD inhibition assay

An *in vitro* XOD inhibitory assay was conducted according to our published method with slight modifications (Xu L. Q. et al., 2021). Firstly, 20 μL XOD stock solution was diluted with 980 μL PBS (pH 7.4) to prepare a working stock enzyme solution. Xanthine powder was dissolved in PBS at a concentration of 7.6 × 10^–2^ mg/ml 50 μL of CP solution (at concentrations of 0.5, 1, 2, 4, 6, 8, 10, 12, and 14 mg/ml), 77 μL of PBS (pH 7.4) and 7 μL of XOD enzyme solution were mixed and incubated at room temperature for 30 min. Then xanthine solution (66 μL) was added to the mixture to initiate the reaction. The reaction mixture was incubated in an oven at 37°C for 30 min, and the UV absorbance was measured at 290 nm. Allopurinol was used as a positive control. The XOD inhibitory activity was determined as follows:

$$XOD\;inhibitory\;activity\;\left(\%\right)=\left[1-\frac{\left(S-S_{0}\right)}{\left(B-B_{0}\right)}\right]\times 100$$

Where S and S_0_ are the absorbances of the reaction system with or without XOD, respectively. B is the absorbance of the reaction system without samples, B_0_ is the absorbance of the reaction system without samples and XOD.

#### Western blotting

Mouse kidney total protein was prepared after homogenization of 100 mg tissues in RIPA buffer with a protease inhibitor cocktail, PMSF, and sodium orthovanadate. Thereafter, supernatants were collected after centrifugation at 12,000 rpm for 10 min at 4°C. The protein concentration was determined using a BCA kit. Equivalent amounts of protein (50 μg/lane) from each group were resolved by 10% SDS-PAGE and then transferred to PVDF membranes by electroblotting. Subsequently, the membranes were blocked in 0.1% TBST containing 5% dried skim milk at room temperature for 1 h, and incubated with the following primary antibodies at 4°C overnight: anti-XOD (1:10,000), anti-ABCG2 (1:1,000), anti-IL-6 (1:1,000), anti-c-Jun (1:1,000), anti-p-c-Jun (1:1,000). Following overnight incubation, primary antibodies were removed and the membranes were rinsed three times with TBST for 10 min each. Next, the membranes were incubated with secondary HRP-conjugated goat anti-rabbit IgG (Immunoglobulin G, 1:4,000) antibody at room temperature for 1 h. Protein bands were visualized by the ECL method and analyzed by ImageJ software (NIH, Bethesda, MA, United States).

#### RT-qPCR

Total RNA from an appropriate amount of preserved kidney and liver was extracted using Trizol reagent (Life Technologies, Inc.). Briefly, tissues (0.10 g) were first homogenized in 1 ml Trizol solution, and the homogenate was mixed with 200 μL chloroform and centrifuged at 12,000 rpm for 15 min at 4°C. The aqueous phase was reserved, precipitated with isopropanol (1:2, v/v), and centrifuged at 10,000 rpm for 10 min. Finally, the RNA was washed with 70% ethanol and resuspended in 20 μL of DEPC water. The quality and concentration of total RNA were measured using a NanoDrop-2000 spectrophotometer. Subsequently, total RNA (1 μg) was subjected to cDNA synthesis using a HiScript II Q RT SuperMix for qPCR (+gDNA wiper) reagent kit (R223-01, Vazyme, Nanjing, China). Real-time PCR was performed with the SYBR Green Master Mix kit (Q711-02, Vazyme, Nanjing, China) according to the manufacturer’s instructions in the CFX 96 Real-Time PCR Detection System (Bio-Rad, CA, United States). The PCR conditions were set as follows: 1 cycle of 95°C for 30 s; 40 cycles of 95°C for 10 s, 60°C for 30 min; 1 cycle of 95°C for 15 s followed by a melting curve step from 65°C to 95°C, 5 s per 0.5°C. The amplification data were evaluated based on the 2^−ΔΔCt^ method, and the results were normalized against *β*-actin. All primers used for RT-qPCR were synthesized by Sangon Biotech Company (Shanghai, China) (Table 1).

**TABLE 1:** Sequences of the primers.

Description	Genebank	Sequence of primers (5′–3′)	Product size (bp)	Tm (◦C)
*XOD*	NM_011723	ATG​ACG​AGG​ACA​ACG​GTA​GAT	185	55.3
		TCA​TAC​TTG​GAG​ATC​ATC​ACG​GT		55.0
*ABCG2*	NM_011920	CAT​CAC​ATC​ACC​TAT​CGA​GTG​A	172	53.7
		CTT​TCC​TTG​CTG​CTA​AGA​CAT​C		53.5
*AKT1*	NM_001331107	ATG​AAC​GAC​GTA​GCC​ATT​GTG	116	55.3
		TTG​TAG​CCA​ATA​AAG​GTG​CCA​T		54.1
*MAPK3*	NM_011952	CAG​CTC​AAC​CAC​ATT​CTA​GGT​A	158	53.9
		TCA​AGA​GCT​TTG​GAG​TCA​GAT​T		53.2
*MAPK8*	NM_001310452	CGC​CTT​ATG​TGG​TGA​CTC​GCT​AC	102	60.5
		CTC​CCA​TGA​TGC​ACC​CAA​CTG​AC		61.0
*TP53*	NM_001127233	ACC​GCC​GAC​CTA​TCC​TTA​CCA​TC	89	61.2
		GGC​ACA​AAC​ACG​AAC​CTC​AAA​GC		59.8
*GAPDH*	NM_001289726	GGT​TGT​CTC​CTG​CGA​CTT​CA	183	57.5
		TGG​TCC​AGG​GTT​TCT​TAC​TCC		56.5

#### Statistical analysis

Data were given as mean ± standard deviation (SD). All datasets were subjected to a normal distribution test first. If they followed normal distribution, one way analysis of variance (ANOVA) followed by Bonferroni post hoc test was used for comparisons, otherwise comparisons were performed using Kruskal–Wallis test. Statistical analyses and graphing were performed using SPSS (Version 19.0, IBM, United States) and GraphPad Prism (Version 6.0, GraphPad Software Inc., CA, United States), respectively. Statistical significance was set at *p* < 0.05.

## Results

### Identification of bioactive compounds and targets

**FIGURE 2:**
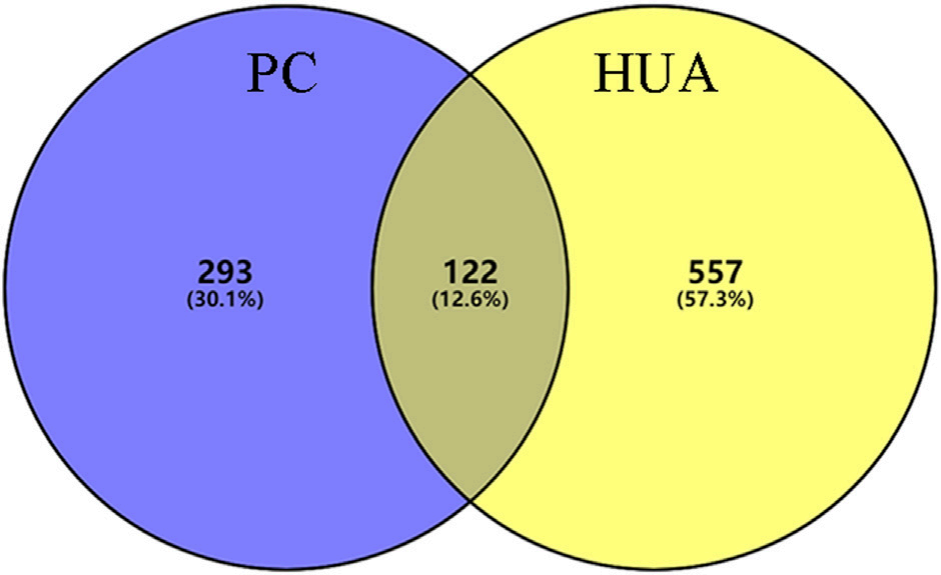
The venn diagram of targets between CP-related targets and HUA-related targets. CP: Cortex Phellodendri; HUA: hyperuricemia.

In this study, 140 compounds were obtained from multiple databases. After screening by ADME parameters (OB ≥ 30%, DL ≥ 0.18, and BBB ≥ −0.3), 27 components were obtained, accounting for 19.29%. Furthermore, 27 components were input into the open-source databases to search for the corresponding targets, among which only 12 compounds were found to be the corresponding targets. These 12 compounds were deemed to be core bioactive components for further analysis, and the detailed information is shown in Table 2. Next, 415 targets interacting with the above 12 core compounds in CP were screened from the TCMSP and GeneCard databases (Supplementary Excel Sheet S1). Furthermore, by screening CTD, OMIM, and DisGeNET databases, 679 HUA-related genes were fished out (Supplementary Excel Sheet S2). Integration of the targets regulated by core compounds and the targets related to HUA revealed that 122 genes overlapped (Figure 2). Therefore, it was reasonable for us to consider that these common genes were the potential therapeutic targets of CP in HUA.

**TABLE 2:** Information of bioactive compounds of CP.

No.	Mol ID	Molecule name	OB (%)	DL	BBB	Caco-2	Structure
1	MOL002663	Skimmianin	40.14	0.20	1.10	1.26	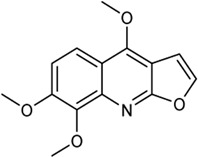
2	MOL000449	Stigmasterol	43.83	0.76	1.00	1.44	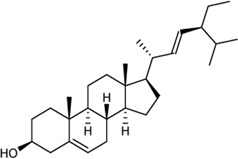
3	MOL000358	β-sitosterol	36.91	0.75	0.99	1.32	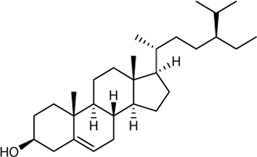
4	MOL005438	Campesterol	37.58	0.71	0.95	1.34	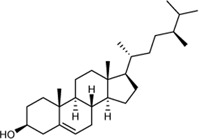
5	MOL002662	Rutaecarpine	40.30	0.60	0.71	1.13	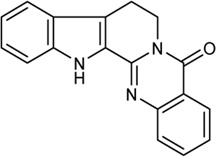
6	MOL001454	Berberine	36.86	0.78	0.57	1.24	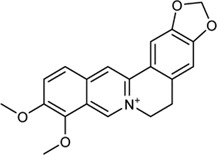
7	MOL002651	Dehydrotanshinone II A	43.76	0.40	0.52	1.02	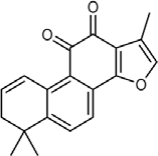
8	MOL002644	Phellopterin	40.19	0.28	0.48	0.98	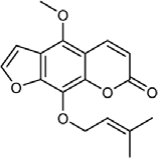
9	MOL000785	Palmatine	64.6	0.65	0.37	1.33	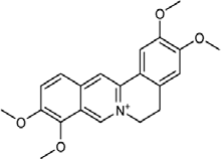
10	MOL001458	Coptisine	30.67	0.86	0.32	1.21	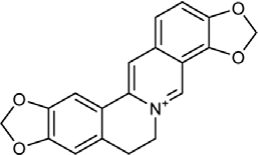
11	MOL002666	Chelerythrine	34.18	0.78	0.28	1.24	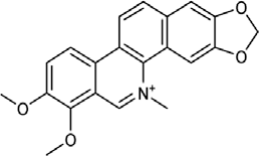
12	MOL002894	Berberrubine	35.74	0.73	0.17	1.07	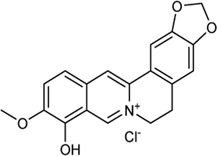

### HCT network construction and analysis

**FIGURE 3:**
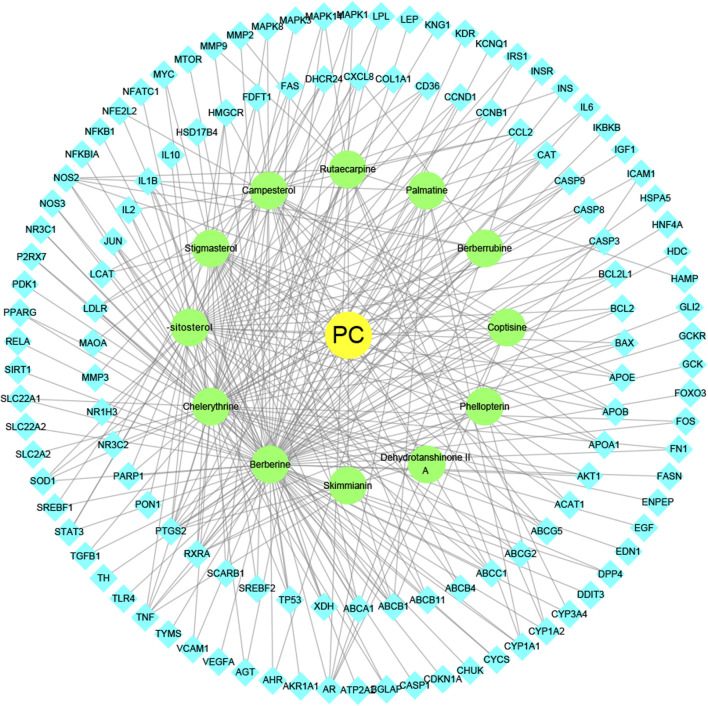
The herb-ingredient-target network of CP. The yellow nodes represent CP, the green nodes represent active herbal ingredients, the cyan nodes represent target proteins, and the lines represent the interactions between them.

To intuitively visualize the interaction correlation between the core bioactive components and the common genes, the HCT network was established by Cytoscape 3.7.1. As shown in Figure 3, the network comprised 489 nodes and 974 interactions. After the network was analyzed by its plugin, the Network Analyzer, components were ranked by degree value in descending order. As shown in Table 3, berberine was found to have the highest degree value (degree = 89), suggesting that berberine might be the most critical ingredient for CP against HUA.

**TABLE 3:** Active ingredients and topological properties.

Compound	Degree	Betweenness	Closeness
Berberine	89	10,799.54	105.83
Chelerythrine	58	4732.711	85.17
β-Sitosterol	34	1927.92	69.17
Stigmasterol	29	2534.62	65.83
Campesterol	24	1154.68	62.50
Rutaecarpine	16	508.88	57.17
Palmatine	11	108.72	53.83
Berberrubine	9	53.73	52.50
Coptisine	6	35.84	50.50
Phellopterin	5	31.78	49.83
Dehydrotanshinone II A	3	5.03	48.50
Skimmianin	2	0.56	47.83

### PPI network construction and analysis

**FIGURE 4:**
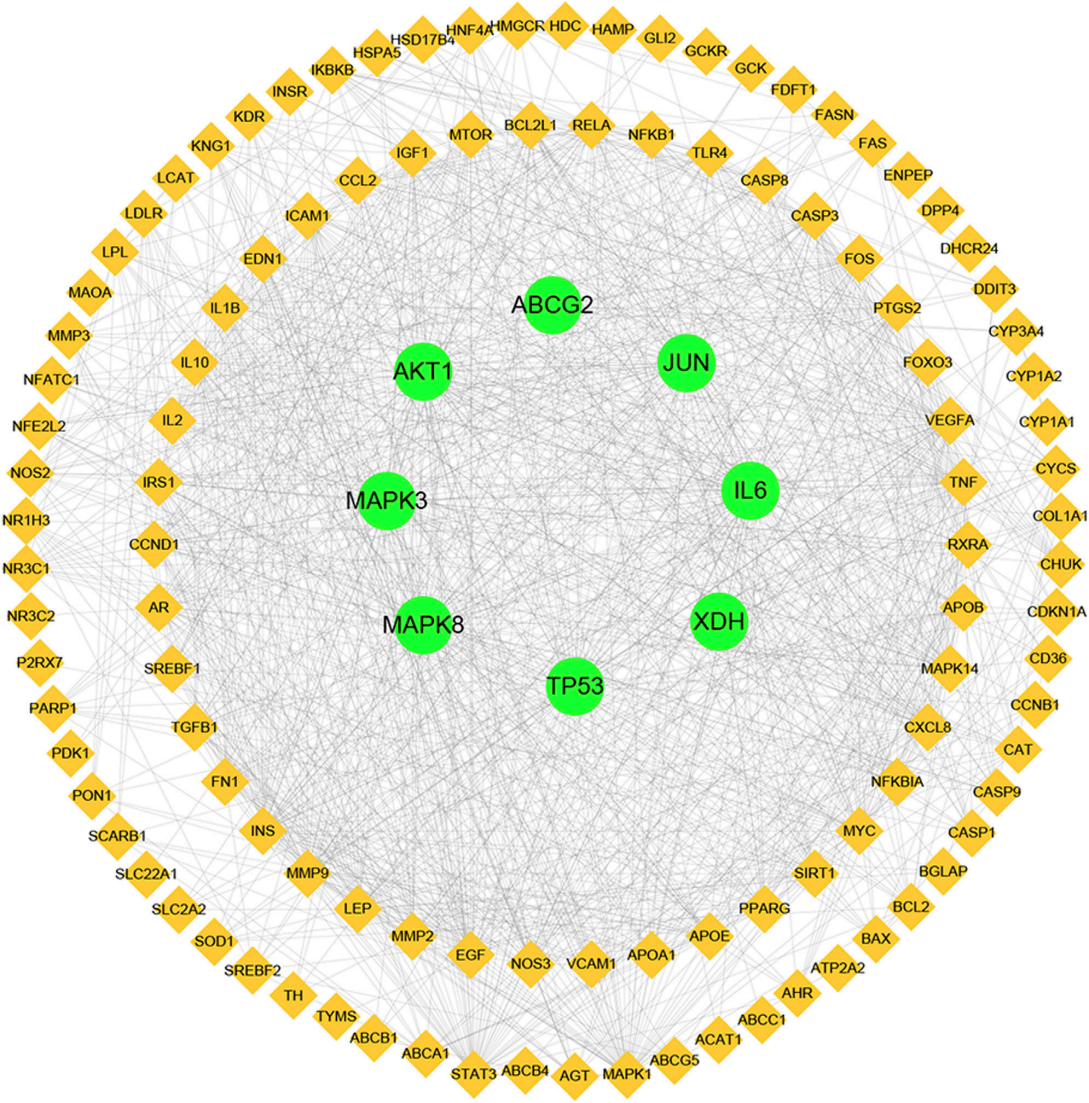
Protein–protein interaction (PPI) network analysis of CP for the treatment of HUA using STRING database.

Next, a PPI network was built using the STRING database and Cytoscape software. As shown in Figure 4, the network comprised 118 nodes and 1079 links. The plug-in “CytoHubba” was used to analyze the topological properties of the PPI network, including degree, closeness centrality, and betweenness centrality (BC). The top 10 genes in each topological algorithm were compared, and the overlapped genes in these three topological parameters were selected as the key genes for further analysis (Table 4).

**TABLE 4:** Hub genes and topological properties.

Gene	Degree	Betweenness	Closeness
*AKT1*	64	0.10572	0.64641
*IL6*	56	0.05602	0.62234
*TP53*	55	0.09232	0.61579
*MAPK8*	55	0.04456	0.60622
*Jun*	53	0.03729	0.59391
*MAPK3*	43	0.02741	0.570732
*ABCG2*	6	0.03560	0.43173
*XDH*	2	0.00063	0.33429

### Enrichment analysis

**FIGURE 5:**
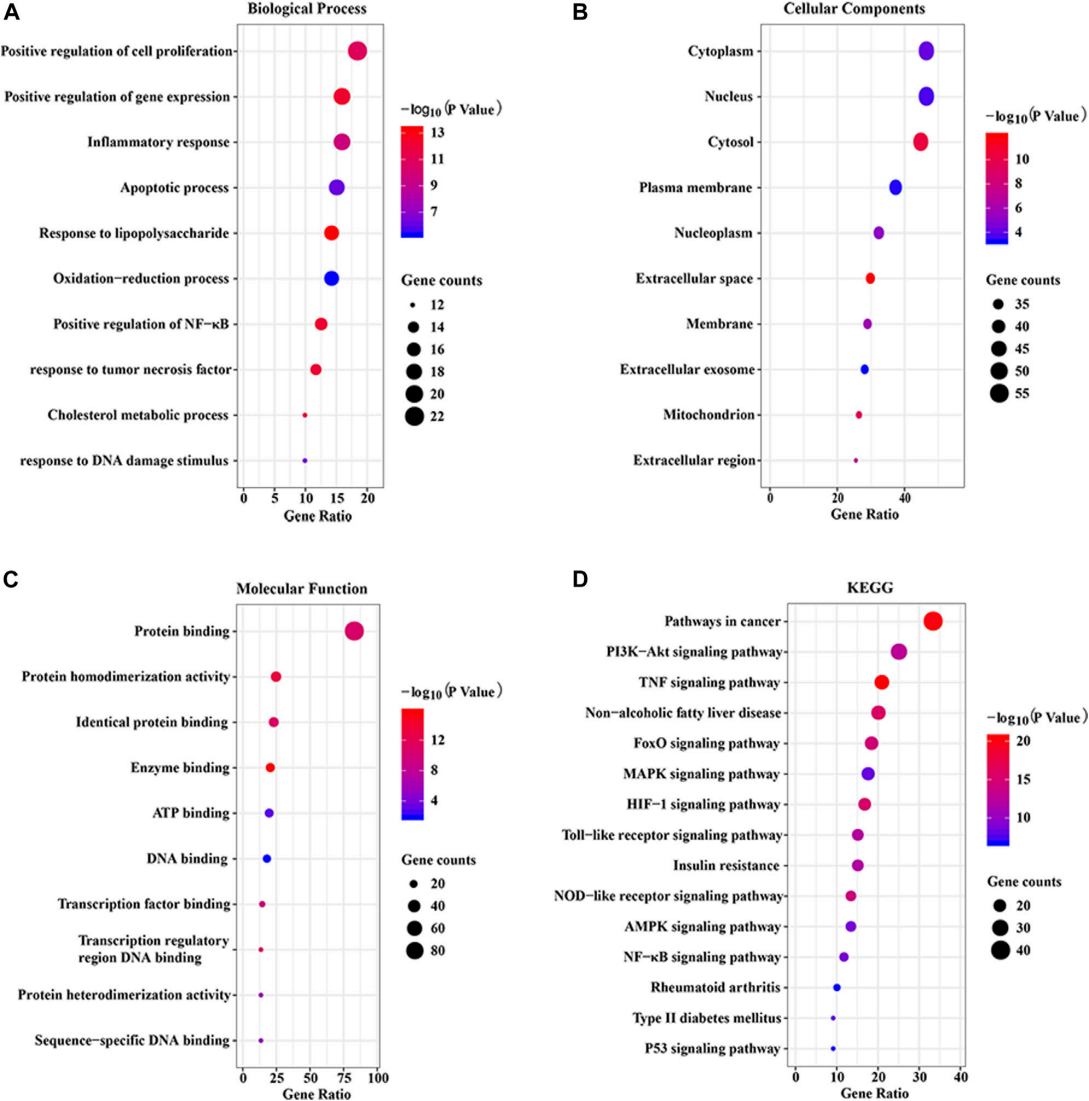
GO and KEGG enrichment analysis of the putative targets. **(A)** The top 10 significantly enriched terms in biological process (BP); **(B)** The top 10 significantly enriched terms in cellular component (CC); **(C)** The top 10 significant enriched terms in molecular function (MF); **(D)** The top 15 significantly enriched terms in KEGG pathway (KEGG).

To explore the potential pharmacological actions of CP, the online database DAVID was utilized to carry out GO and KEGG annotation. The top 10 significantly enriched GO terms and top 15 KEGG pathways were plotted as bubble charts using the R package ggplot2. As shown in Figure 5, the biological process (BP) was significantly enriched in positive regulation of cell proliferation and gene expression, inflammatory response, and apoptotic process, etc. Cellular component (CC) was significantly enriched in cytoplasm, nucleus, cytosol, plasma membrane, and nucleoplasm, etc. Molecular function (MF) was significantly enriched in protein binding and homodimerization activity, identical protein binding, enzyme binding, and ATP binding, etc. Moreover, KEGG enrichment analysis indicated that 122 overlapping genes were significantly enriched in inflammation and apoptosis signaling pathways, including PI3K/AKT, TNF, FoxO, MAPK, HIF-1, TLR, NLRP3, AMPK, and NF-κB, etc.

### CTP network construction and analysis

**FIGURE 6:**
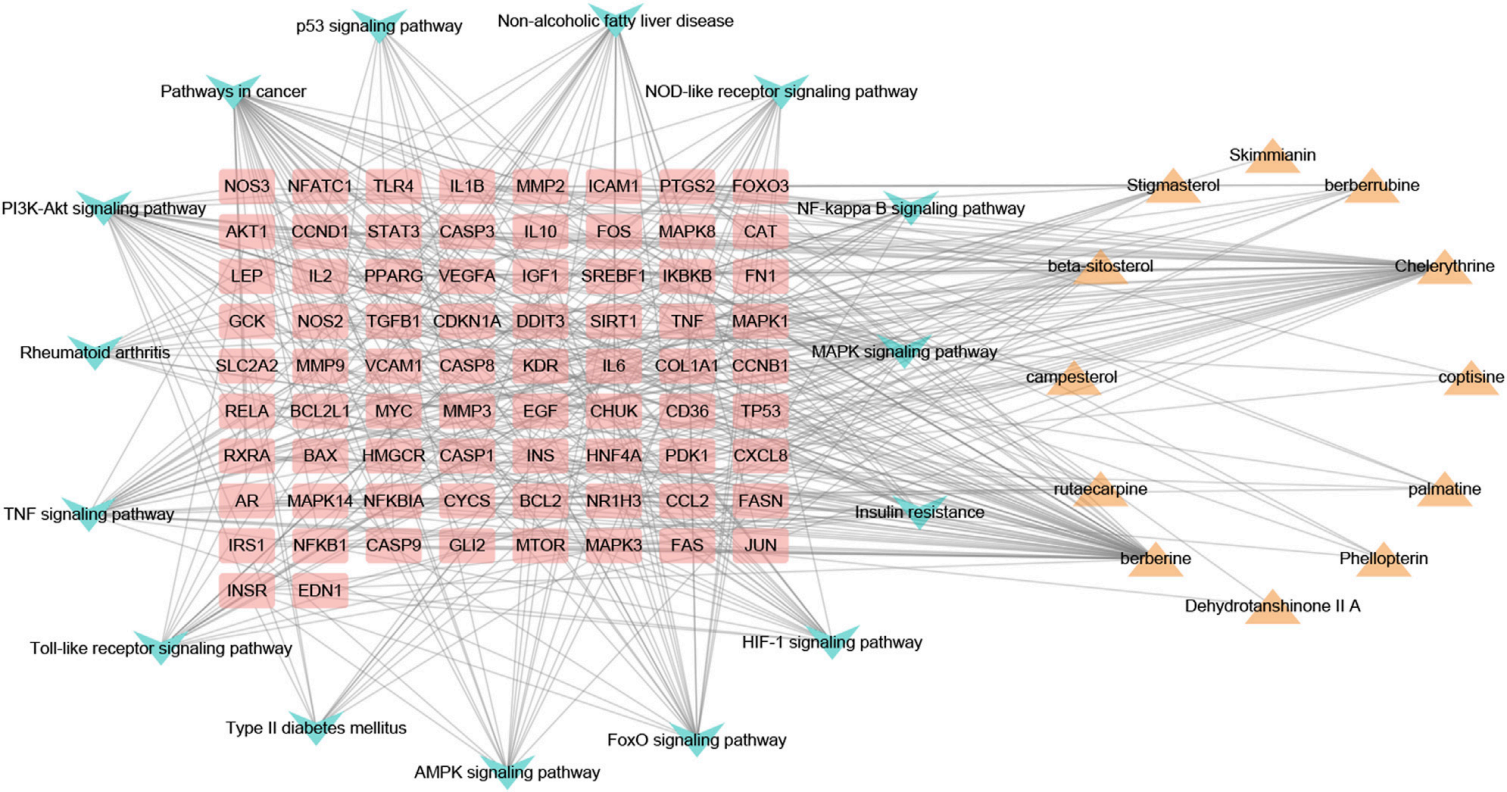
The component-target-pathway network of CP. Yellow triangle nodes represent active ingredients, pink rectangles represent nodes target proteins, blue arrowhead nodes represent pathways, and the lines represent the interactions between them.

As shown in Figure 6, the CTP network comprised 101 nodes and 468 links. According to the network analysis, one pathway and compound could act on multiple targets, and one target could act on multiple pathways and compounds. This network diagram fully reflected the characteristics of the synergistic relationship between multiple components, targets, and pathways of CP treatment of HUA.

### Effects of CP on body weight and organ index

No deaths were observed in any mice during the course of the study. No significant differences were seen in body weight, liver weight, or liver index among the experimental groups (Table 5). While kidney and spleen weights and indexes were significantly higher in the HUA group than those in the NC group (*p* < 0.05/0.01). CP treatment dramatically decreased the kidney weight and kidney indexes (*p* < 0.01). However, CP did not cause any significant reduction in the spleen weight or spleen index.

**TABLE 5:** Effect of CP on body weight, organ weight and organ index.

Group	Body weight (g)	Organ weight (g)			Organ index (%)		
		Liver	Kidney	Spleen	Liver	Kidney	Spleen
NC	27.80 ± 2.92	1.37 ± 0.11	0.30 ± 0.07	0.067 ± 0.016	4.96 ± 0.52	1.06 ± 0.14	0.24 ± 0.05
HUA	27.14 ± 3.20	1.41 ± 0.17	0.53 ± 0.07##	0.091 ± 0.017#	5.22 ± 0.58	1.97 ± 0.38##	0.34 ± 0.06##
Feb (5)	28.78 ± 2.35	1.46 ± 0.11	0.32 ± 0.04**	0.081 ± 0.014	5.08 ± 0.34	1.10 ± 0.14**	0.28 ± 0.05
CP (200)	28.23 ± 2.36	1.48 ± 0.12	0.32 ± 0.06**	0.095 ± 0.014	5.23 ± 0.49	1.12 ± 0.24**	0.34 ± 0.03
CP (400)	28.85 ± 2.40	1.44 ± 0.13	0.29 ± 0.03**	0.095 ± 0.011	5.12 ± 0.61	1.01 ± 0.16**	0.33 ± 0.04
CP (800)	28.96 ± 2.20	1.45 ± 0.18	0.28 ± 0.05**	0.100 ± 0.020	4.96 ± 0.56	0.97 ± 0.20**	0.35 ± 0.07

The results are expressed as mean ± SD (*n* = 10). ^#^ *p* < 0.05, ^##^ *p* < 0.01 comp/ared with NC, group; ^*^ *p* < 0.05, ^**^ *p* < 0.01, compared with HUA, group.

### Effects of CP on liver UA, serum UA, cre, and BUN levels

**FIGURE 7:**
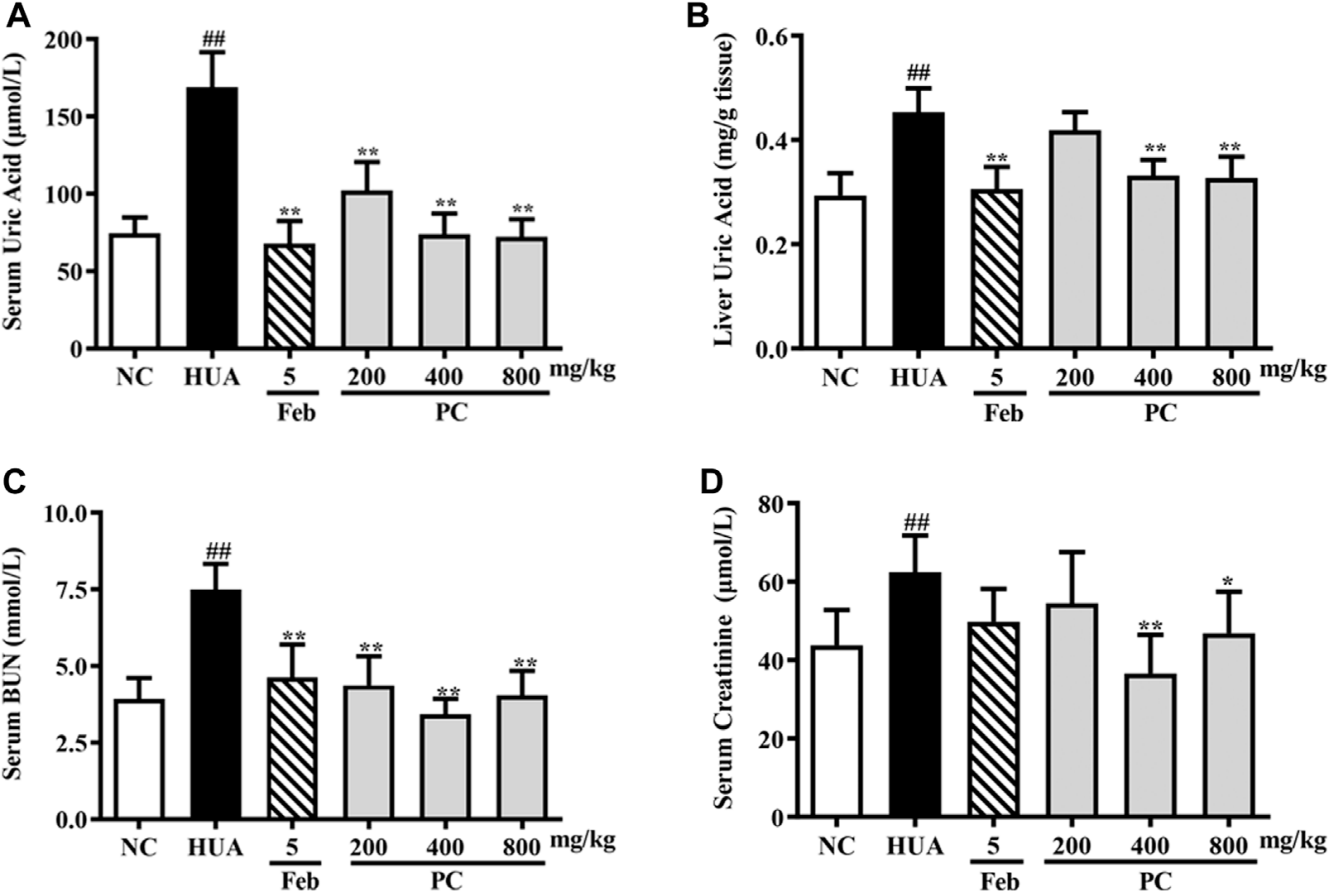
Effects of CP on liver UA, serum UA, Cre, and BUN levels in PO/HX-induced HUA mice. **(A)** The serum level of UA. **(B)** The liver level of UA. **(C)** The serum level of BUN. **(D)** The serum level of Cre. The results are expressed as mean ± SD (*n* = 10). ^##^ *p* < 0.01 compared with NC group; ^*^ *p* < 0.05, ^**^ *p* < 0.01 compared with HUA group.

As indicated in Figure 7A, compared to the NC group, a nearly 126.7% increase in sUA level was noted in HUA mice induced by HX combined with PO (*p* < 0.01), indicating the HUA model was successfully constructed. CP and Feb treatment remarkably decreased the sUA level in HUA mice (*p* < 0.01) Feb decreased the sUA level by 59.8% and three-dose CP groups decreased the sUA level by approximately 39.5%, 56.3%, and 57.3%, respectively.

In addition, as shown in Figure 7B, PO/HX induced a remarkable elevation in liver UA levels in mice compared to the NC group mice (*p* < 0.01). Feb, a positive control drug, significantly decreased UA level in the liver compared with the HUA group (*p* < 0.01). Compared with the HUA group, the UA level in the liver was significantly reduced by CP at doses of 400 and 800 mg/kg (*p* < 0.01), respectively.

As shown in Figures 7C,D, compared with the NC group, the serum Cre and BUN levels in the HUA group were observably increased by 42.6% and 90.9%, respectively (*p* < 0.01). Nonetheless, both serum Cre and BUN levels were remarkably reduced after CP treatment (*p* < 0.05/0.01), indicating that CP could alleviate kidney injury in HUA mice.

### XOD inhibition assay *in vitro* and *in vivo*

**FIGURE 8:**
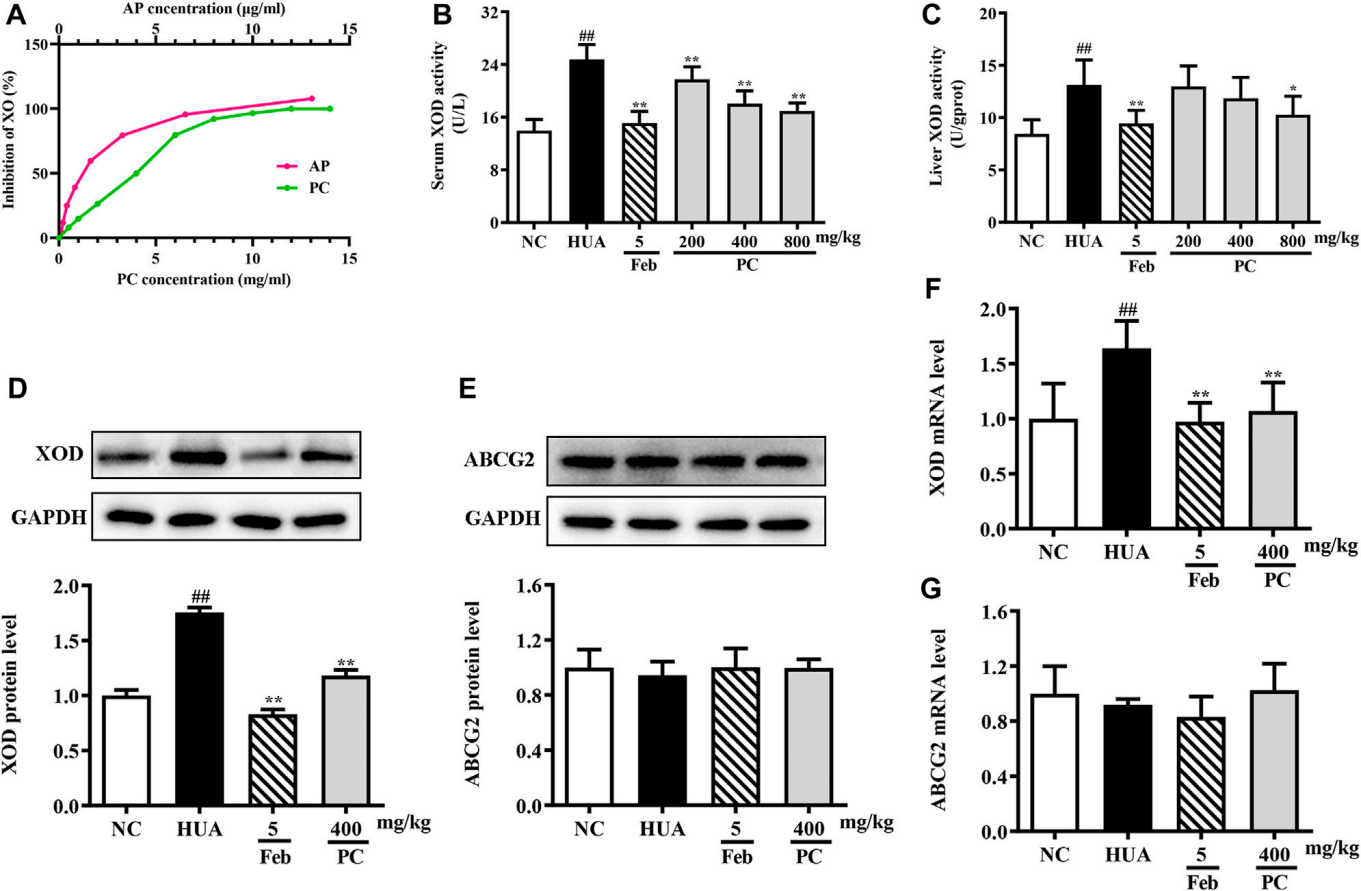
Effects of CP on XOD activity and ABCG2 expression. **(A)** *In vitro* XOD inhibition. **(B)** Serum XOD activity. **(C)** Liver XOD activity. **(D)** Relative protein expression levels of hepatic XOD. **(E)** Relative protein expression level of renal ABCG2. **(F)** Relative mRNA level of hepatic XOD. **(G)** Relative mRNA level of renal ABCG2. The protein expression level was determined by Western blotting and the relative gene level was determined by RT-qPCR. The results are expressed as mean ± SD (*n* = 10). ^##^ *p* < 0.01 compared with NC group; ^*^ *p* < 0.05, ^**^ *p* < 0.01 compared with HUA group.

As shown in Figure 8A, the activity of XOD was inhibited by CP extract *in vitro* in a dose-dependent manner, and the IC_50_ of CP extract was 3.34 mg/ml. While the IC_50_ of the positive control, allopurinol, was 1.08 × 10^–3^ mg/ml.

*In vivo*, compared to the NC group, the serum and liver XOD levels in the HUA group were significantly increased by 77.3% (*p* < 0.01) and 55.3% (*p* < 0.01), respectively (Figures 8B,C). However, CP administration effectively decreased serum XOD activity by approximately 12.2%, 27.0% and 31.5% (all *p* < 0.05), respectively. And the mice treated with 800 mg/kg of CP exhibited a significant reduction in liver XOD activity by 21.7% (*p* < 0.05).

The XOD mRNA and protein levels were assessed via RT-qPCR and Western blot analyses. Compared to the NC mice, the relative expression of XOD protein (Figure 8D) and mRNA (Figure 8F) was significantly increased in the HUA mice (all *p* < 0.01), while CP administration distinctly reversed these effects (*p* < 0.01). As expected, Feb also significantly reduced the mRNA and protein levels of XOD in HUA mice.

### Effects of CP on renal ABCG2 expression level

The mRNA and protein expression of ABCG2 in the kidney was assessed. As revealed in Figure 8E &G, no differences were observed among the experimental groups.

### Effects of CP on kidney histopathological changes

**FIGURE 9:**
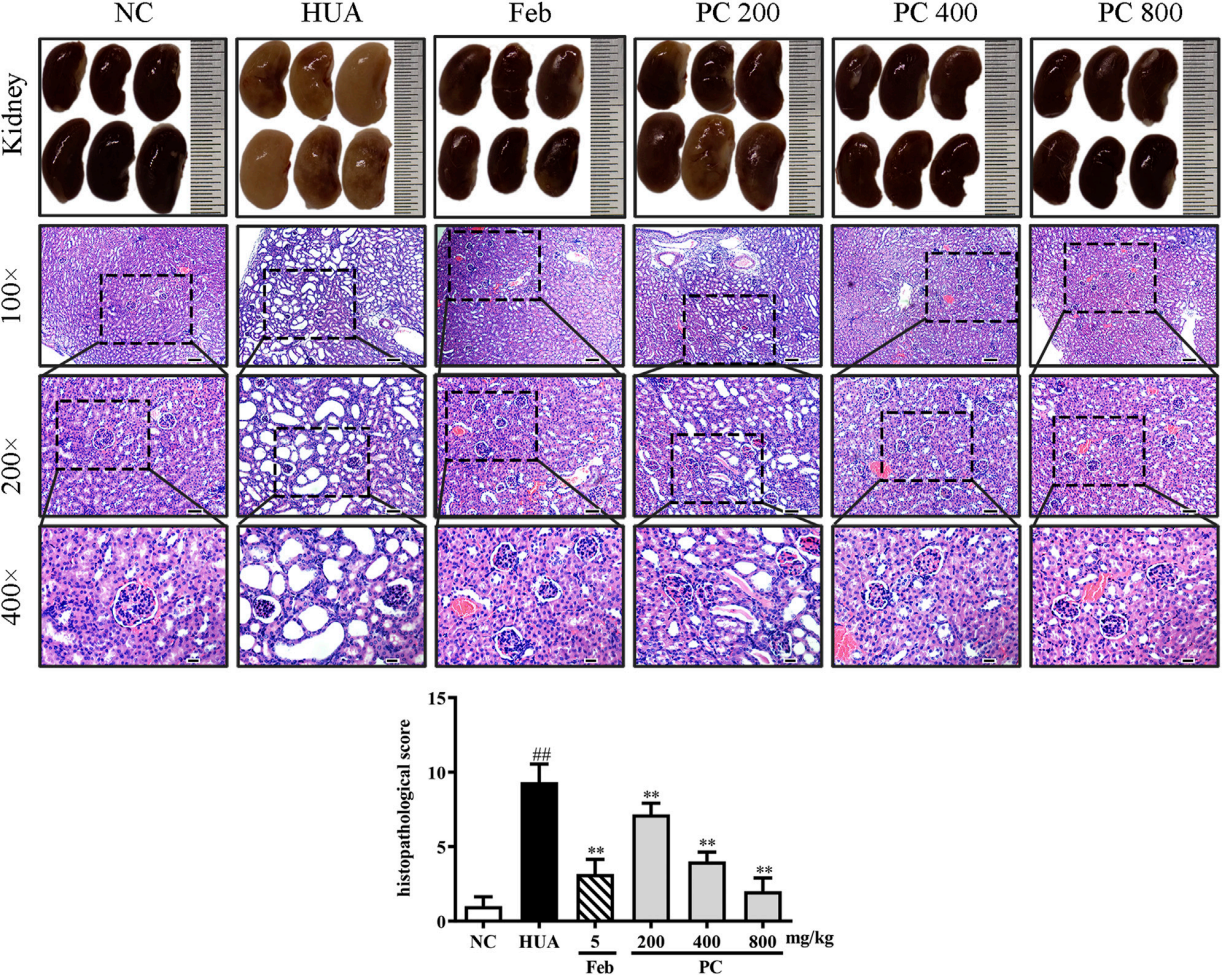
Effects of CP on kidney general status and histopathological changes in PO/HX-induced HUA mice. Gross appearance of the kidneys from all six groups were photographed, and sections were subjected to H&E staining; these representative photographs were taken at a magnification of 100 × (scale bar 100 μm), 200× (scale bar 50 μm), 400× (scale bar 20 μm), respectively.

In Figure 9, the kidneys from the NC group showed a reddish, shiny, and elastic appearance. HE staining results indicated that the kidney in the NC group presented a normal architecture with an intact glomerulus and renal tubules. However, the gross appearance of the kidney in the HUA group was pale and irregularly shaped. HE-stained kidney sections of the HUA model group also revealed a marked renal injury, as manifested by severe tubular dilation, mild inflammatory cell infiltration, renal tubules with protein casts, swelling, and proximal tubule necrosis. Feb and CP administration effectively mitigated the renal injury, and the renal protective effect of CP was dose-dependent. The histopathological scores of kidneys in the HUA group were significantly higher than those in the NC group (*p* < 0.01). The histopathological scores of the kidney from mice in Feb and PC groups were significantly lower than those from mice in the model group (*p* < 0.01).

### Effects of CP on the expression of hub genes

**FIGURE 10:**
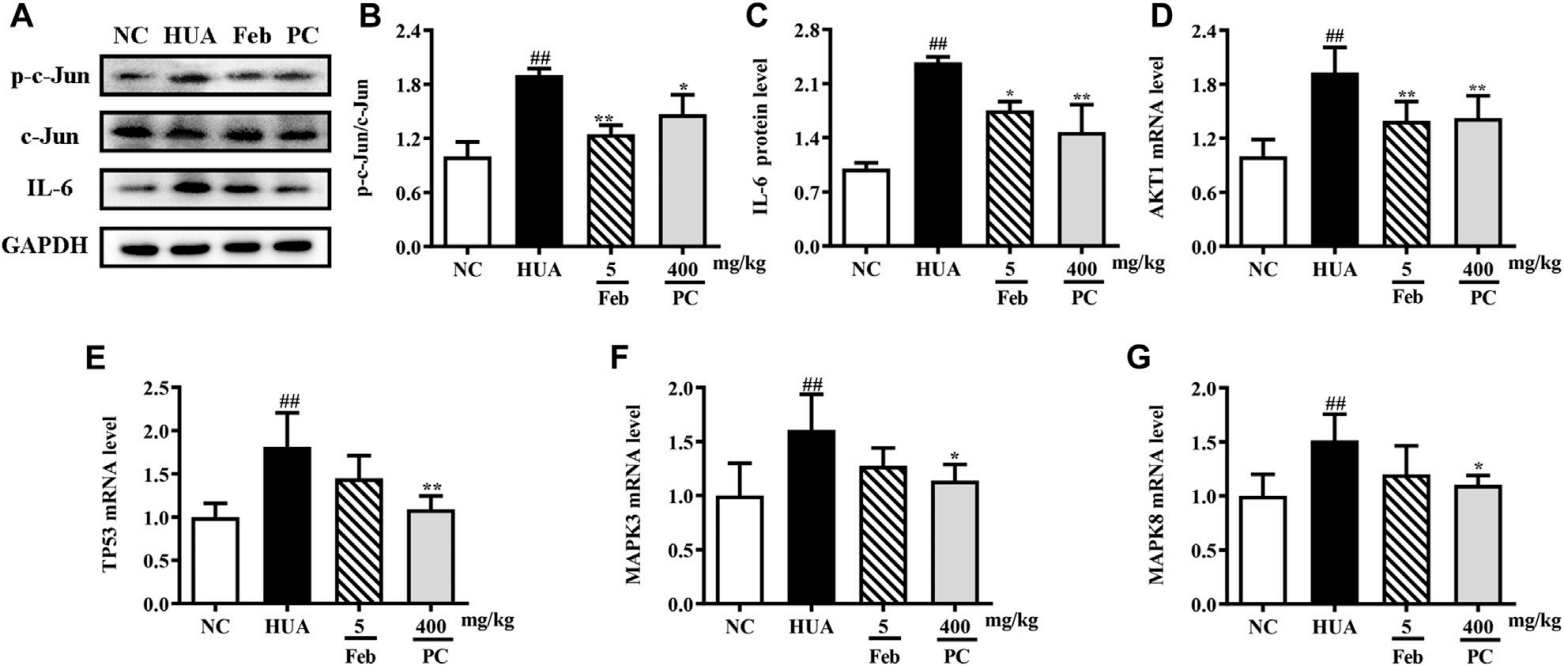
Effects of CP on the expression of hub genes. **(A)** Representative western blot bands. Relative protein expression levels of **(B)** p-c-Jun/c-Jun and **(C)** IL-6. Relative mRNA expression levels of **(D)** AKT1, **(E)** TP53, **(F)** MAPK3, and **(G)** MAPK8. The results are expressed as mean ± SD. ^##^ *p* < 0.01, compared with NC group; ^*^ *p* < 0.05, ^**^ *p* < 0.01, compared with HUA group.

To further validate the effects of CP on the hub genes predicted by network analysis, the mRNA and protein expression levels were detected. As shown in Figure 10, the protein expression levels of p-c-Jun and IL-6, as well as the mRNA levels of *MAPK3*, *MAPK8*, *AKT*, and *TP53* were markedly up-regulated in the kidney tissues of HUA mice than in those of the NC mice (*p* < 0.01). Notably, CP treatment succeeded in restoring HUA-induced over-expression of the hub genes.

## Discussion

HUA is a metabolic abnormality caused by urate overproduction, urate underexcretion, extra-renal urate excretion caused by ABCG2 dysfunction, or both (Ichida et al., 2012; Dalbeth et al., 2016). To date, genome-wide association studies (GWAS) have found various genetic loci that influence sUA levels, such as *GLUT9*, *URAT1*, *NPT1*, and *ABCG2* (Nakatochi et al., 2019). These genes are validated targets for several existing drugs that have been widely used in clinical practice for treating HUA and gout. Allopurinol and febuxostat are xanthine oxidase inhibitors (XOIs) that block the formation of UA and are recommended as the first-line pharmacologic urate-lowering agents. However, allopurinol can induce severe hypersensitivity syndrome (AHS) (Perez-Ruiz and Dalbeth, 2019). Febuxostat has been reported to be more effective at a fixed or limited dose (maximum 200–300 mg/day) in clinical trial, however accompanied by major adverse effects such as abnormal liver function and gout flares (Becker et al., 2005; Becker et al., 2009). Benzbromarone and lesinurad are considered the second-line ULTs for gout that lower sUA levels by inhibiting URAT1. However, lesinurad was not available in the United States due to a high incidence of renal side effects (Jansen et al., 2018), and benzbromarone is not licensed in the United States due to concerns about its hepatotoxicity (Lee et al., 2008). Therefore, the development of anti-HUA drugs with high efficiency and mild or no side effects is badly needed. CP has been used as a core herb in China for the treatment of HUA for thousands of years. In the present study, network pharmacology methods and experimental validations were performed to further explore the potential mechanisms and therapeutic effects of CP on HUA for the first time. These findings laid the foundation for future clinical research in this area.

The serum UA level has been widely accepted as a reliable indicator of HUA, and the liver is the most critical organ for UA production. Hence, in this study, the serum and liver UA levels were determined. Our results revealed that administration with CP obviously attenuated the rise of UA levels in serum and liver. Besides, many studies have suggested that a high level of serum UA is closely related to renal dysfunction (Akioka et al., 2017). Serum BUN and Cre are the two most widely used indexes to assess renal function. Besides, several parameters, including kidney weight and index, and renal histopathology and morphology, were evaluated for the protective effect of CP against HUA-induced renal toxicity. Results suggested that CP exerted promising nephroprotective effects in HUA mice, as evidenced by reduced kidney weight and index, decreased serum BUN and Cre levels, alleviated histopathological alterations, as well as normalized kidney morphology. Conclusively, CP exhibited remarkable ameliorative effects against PO/HX-induced HUA and renal injury.

In this study, 12 bioactive compounds of CP were screened out from the TCMSP database, 415 CP-related targets were selected from the TCMSP and Gene Cards databases, and 679 HUA-related genes were obtained from the OMIM, CTD, and DisGeNET databases. Then a total of 122 herb and disease co-target genes were obtained and adopted to establish an “HCT” network. In the network, the key bioactive ingredients such as berberine (degree 89), chelerythrine (degree 58), *β*-sitosterol (degree 34), stigmasterol (degree 29), campesterol (degree 24), rutaecarpine (degree 16), palmatine (11), and berberrubine (degree 9) had a high degree value. Berberine (BBR) is a naturally occurring isoquinoline alkaloid and the main effective compound of Phellodendri Cortex. Berberrubine (BRB) is deemed as one of the main metabolites of BBR. Our previous study has indicated that BBR and BRB effectively lowered the sUA levels in PO/HX-induced HUA mice (Lin et al., 2021; Shi et al., 2021). Chelerythrine is a natural benzo[c]phenanthridine alkaloid derived from *Chelidonium majus*, *Macleaya cordata*, and *Sanguinaria canadensis* (Wu et al., 2018). It possesses anti-tumor (Zhang et al., 2011), anti-diabetes (Zheng et al., 2015), and anti-inflammatory properties (Niu et al., 2011), and protective effects against ethanol-induced gastric ulcer (Li et al., 2014) and lipopolysaccharide-induced endotoxic shock (Niu et al., 2014). Besides, chelerythrine is also beneficial for obstructive nephropathy (Juan et al., 2012; Shi et al., 2019). In most of the cases, *β*-sitosterol, campesterol, and stigmasterol are among the most common phytosterols, which possess diverse pharmacological properties, including anti-cancer, anti-inflammatory, anti-oxidant, and blood lipid-lowering activities (Trautwein and Demonty, 2007). It has been reported that *β*-sitosterol is able to alleviate monosodium urate (MSU) crystal-induced paw oedema in mice (de Souza et al., 2012). Previous studies have demonstrated that stigmasterol can reduce sUA levels in HUA mice via inhibiting XOD activity and remarkably decrease the paw edema induced by MSU crystals (Ferraz-Filha et al., 2016). To our knowledge, the hypouricemic effects of rutaecarpine and palmatine have yet to be investigated. However, these compounds have been reported to show anti-inflammatory effects (Luo et al., 2020; Ma et al., 2021). Collectively, these findings suggest that CP displayed anti-HUA action via multiple ingredients that acted on multiple targets.

To further elucidate the molecular mechanisms, a PPI network containing 118 nodes and 1079 links was established using the common potential targets of CP and HUA. Then topological property analysis was performed, and the top 6 genes obtained by calculating the degree, betweennes, and closeness centrality algorithms were selected as hub genes: *TP53*, *MAPK8*, *MAPK3*, *IL-6*, *c-Jun*, and *AKT1*. However, due to the strict screening criteria, XOD and ABCG2, two common intersection targets of CP and HUA, were excluded. In view of their important roles in UA metabolism, the expression of XOD and ABCG2 were also evaluated in this study. XOD is a key enzyme that catalyzes hypoxanthine into xanthine and then metabolizes xanthine to UA. ABCG2 is a high-capacity transporter that facilitates UA excretion in the intestine and kidney (Woodward et al., 2009). In this study, CP exerted inhibitory activity on XOD *in vitro* by direct assay and *in vivo* by using a HUA mouse model. However, no significant difference was observed in kidney protein and mRNA levels of ABCG2 among all groups, whereas the levels of ABCG2 in intestinal samples were not examined and need to be explored further. Consistent with the foregoing result, XOD is a key target for CP to lower UA (Kong et al., 2004). IL-6 acts mostly as a proinflammatory cytokine involved in the pathogenesis of gout, and may be a reliable prognostic marker for patients with gout (Cavalcanti et al., 2016). *TP53* (also known as p53) is a multi-functional tumor suppressor gene and functions as a transcriptional regulator that governs multiple cellular processes, such as cell cycle, DNA repair, apoptosis, and senescence. Previous studies have demonstrated that p53 may be a key modulator of IL-6 in the synovium and plays a prominent role in the inhibition of inflammation (Zhang et al., 2016). The most common proto-oncogene, c-Jun, is activated by various extracellular signals and serves important roles in cell proliferation, differentiation, apoptosis, and invasion. And the activation of c-Jun is also closely related to the regulation of inflammation and/or fibrosis in human renal disease (De Borst et al., 2007). Akt1 is a crucial downstream effector of PI3K/Akt signaling and plays a role in the transcriptional modulation of a subset of genes associated with cell growth, proliferation, and apoptosis (Hixon and Boekelheide, 2003). Previous studies have reported that inhibition of activity of the Akt1 protein could decrease the deposition of fibronectin and improve diabetic nephropathy (Hong et al., 2013). MAPK3 (also known as ERK1) and MAPK8 (also known as ERK1) are both mitogen-activated protein kinases that can mediate the progress of cell growth, differentiation, inflammatory response, proliferation, apoptosis, and other pathological processes. This study found that CP significantly inhibited the mRNA and protein expression of hepatic XOD in the HUA mice, as well as the expression of renal TP53, MAPK8, MAPK3, IL-6, c-Jun, and Akt1 at mRNA or protein level, which was in line with the network pharmacology analysis results. However, no significant changes in the mRNA and protein expression levels of ABCG2 by CP treatment were shown in HUA mice. And the expression of ABCG2 in the small intestine needs further study.

Following the KEGG signal pathway analysis, the mechanisms of CP in the treatment of HUA mainly involved inflammation and apoptosis signaling pathways, including PI3K/Akt, TNF, FoxO, MAPK, HIF-1, TLR, NLRP3, AMPK, and NF-κB. The PI3K/Akt pathway is a crucial pathway that regulates cell proliferation, cell survival, cell cycle progression, and cancer metabolism (Ocklenburg et al., 2021). Studies have found that in the Uox-knock-out-induced HUA and nephropathy rat line, the PI3K inhibitor 3-MA alleviates kidney injury accompanied by renal fibrosis, macrophage infiltration, and expression of NLRP3 and IL-1β in injured kidneys (Wu et al., 2021). Furthermore, soluble UA regulates the PI3K/Akt pathway and TLR4-NLRP3 inflammasome and increases the expression of ABCG2 and PDZK1, therefore accelerating the excretion of UA in the intestines and feces (Chen et al., 2018). Emerging evidence has indicated that the NLRP3 inflammasome plays a crucial role in the progression of HUA and kidney inflammation. And our previous study has indicated that dihydroberberine, one of the main metabolites of BBR in the intestinal ecosystem, ameliorates HUA and inflammation by blocking the NLRP3 inflammasome activation in PO/HX-induced hyperuricemic mice (Xu L. Q. et al., 2021). Extracellular UA and MSU have been recognized by TLR2 and TLR4 in gouty arthritis and activate the MAPK, NLRP3 and NF-κB signaling pathways, thus initiating the inflammatory response (Qing et al., 2014; Xiao et al., 2015). Literature information suggests that Cortex Phellodendron exerts crucial anti-inflammatory effect by inhibiting NF-κB and MAPK pathways (Choi et al., 2014). In conclusion, CP exerted anti-HUA effect by modulating multiple signaling pathways.

## Conclusion

In the present work, a network pharmacology approach combined with *in vitro* experimental validation was integrated to investigate the bioactive ingredients, targets, and pathways of CP against HUA. Our findings indicated that CP exerted potent anti-HUA effect by inhibiting UA production, and possessed nephroprotective effects, at least in part, by inhibition of inflammatory and apoptotic pathways, e.g., PI3K/AKT, TNF, MAPK, TLR, and NLRP3. Although we have used a network pharmacology approach to preliminarily analyze the hub genes involved in the therapeutic effect of CP against HUA, their specific regulation mechanism merits further investigation.

## Data Availability

The original contributions presented in the study are included in the article/Supplementary Material, further inquiries can be directed to the corresponding authors.
