# Supplementary material for: Integrating network pharmacology and experimental validation to clarify the anti-hyperuricemia mechanism of cortex phellodendri in mice
Source: Front Pharmacol. 2022 Nov 10;13:964593. doi: 10.3389/fphar.2022.964593 (PMC9692208; doi:10.3389/fphar.2022.964593)

**Original images for western blots**

From left to right are the NC group, HUA group, Feb group, PC 200, PC 400, PC 800, [respectively](javascript:;)

Liver XOD


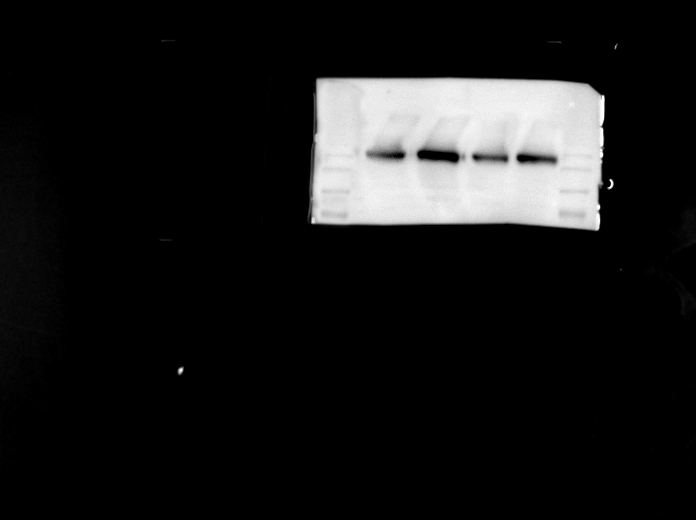

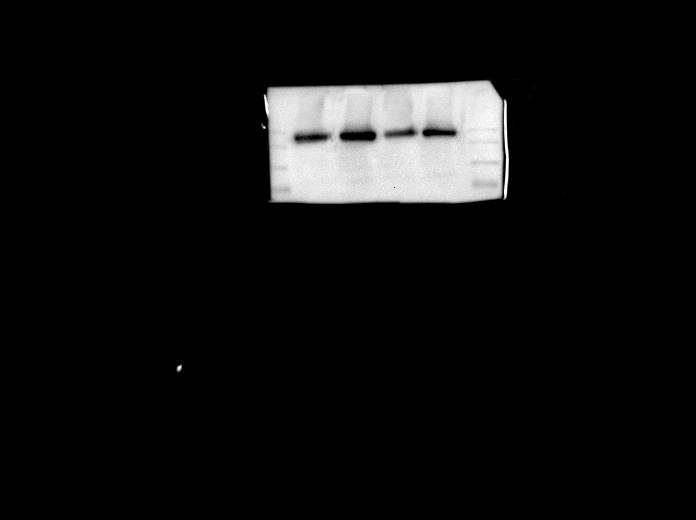

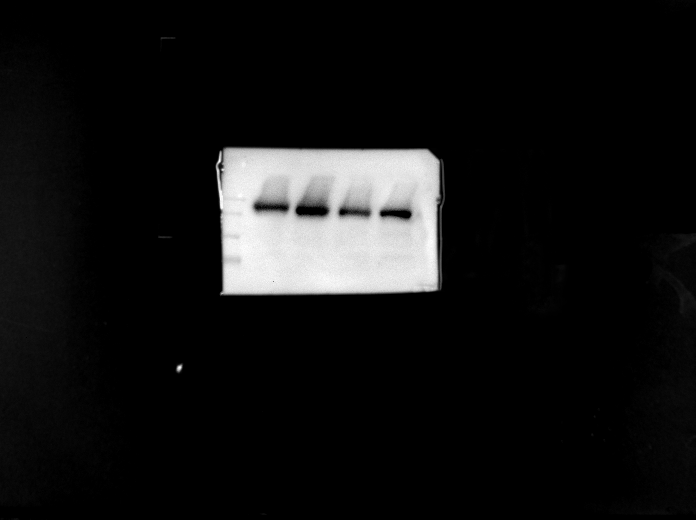


Liver GAPDH


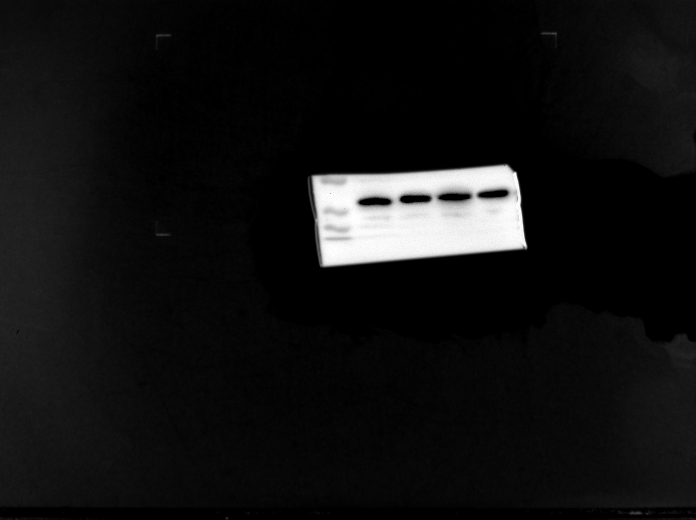

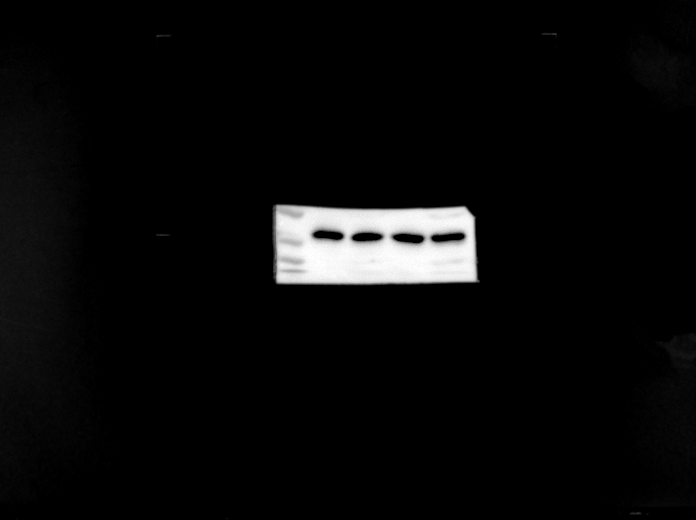

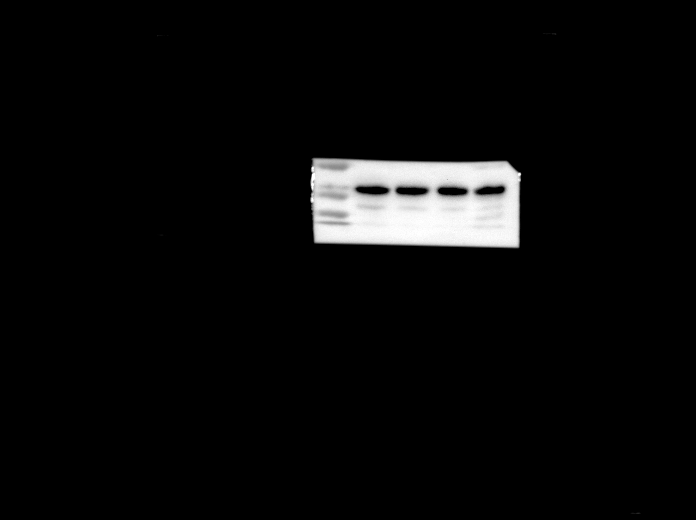


Kidney ABCG2


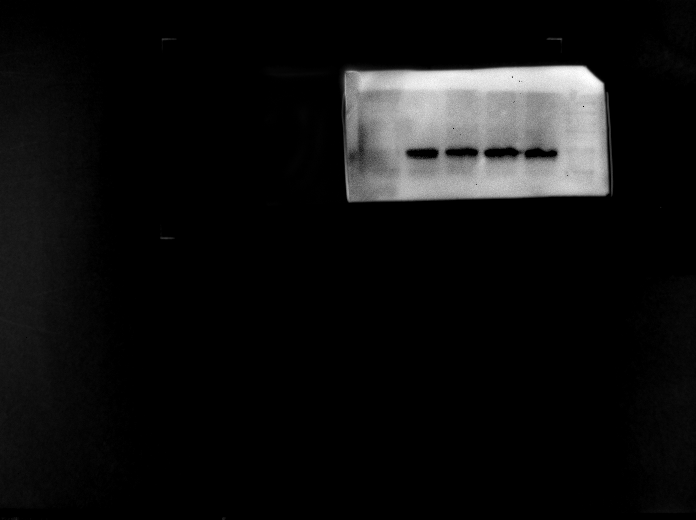

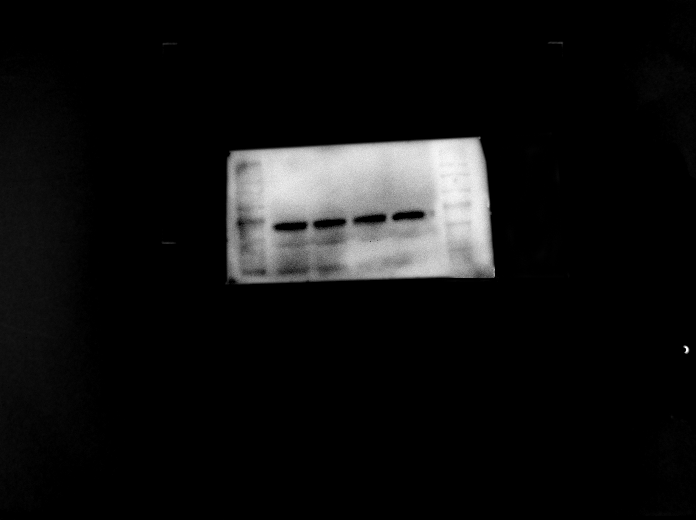

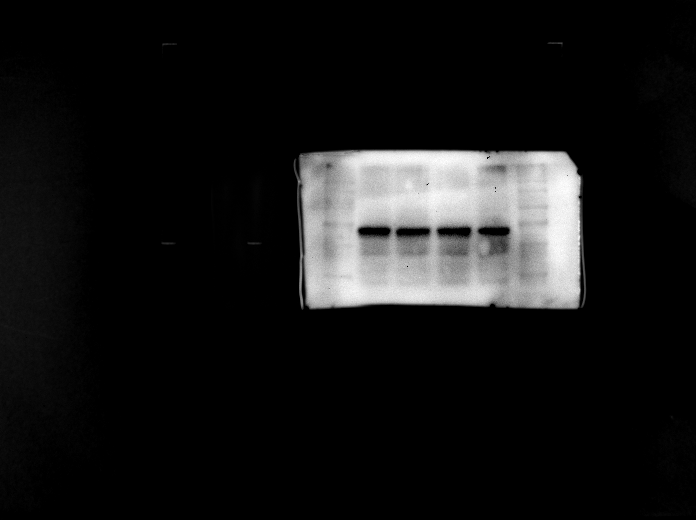


Kidney IL-6


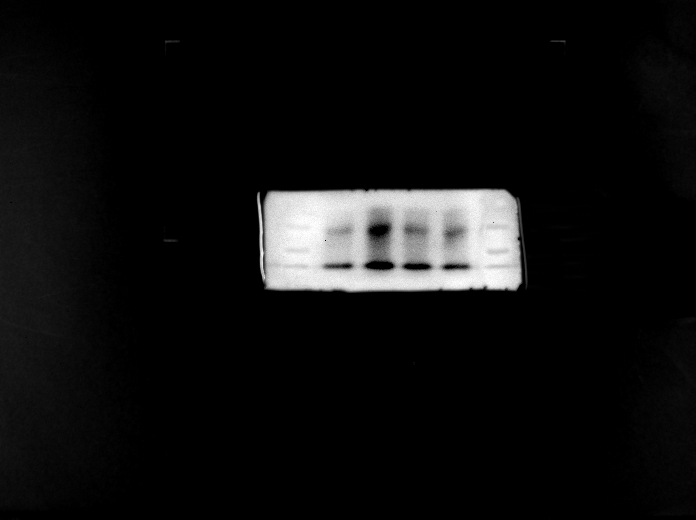

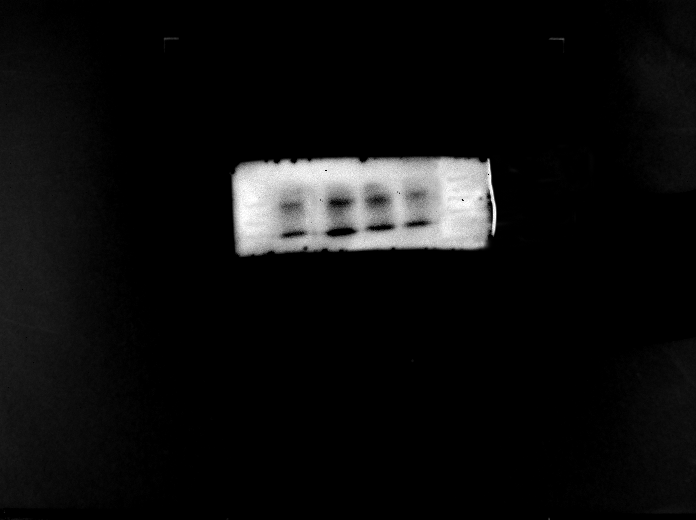

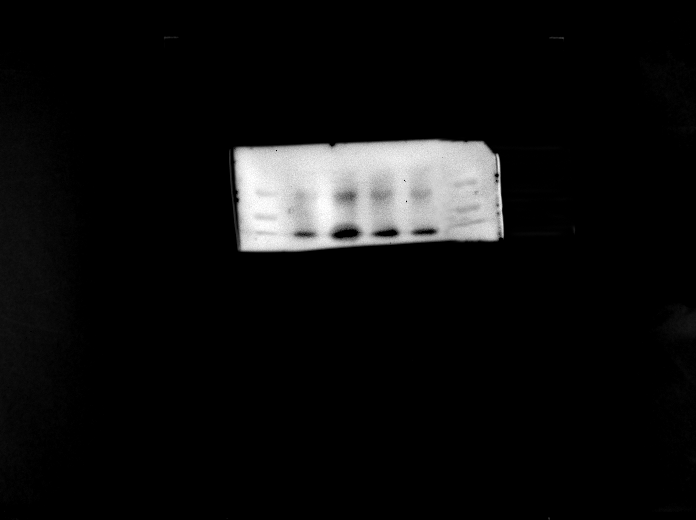


Kidney c-jun


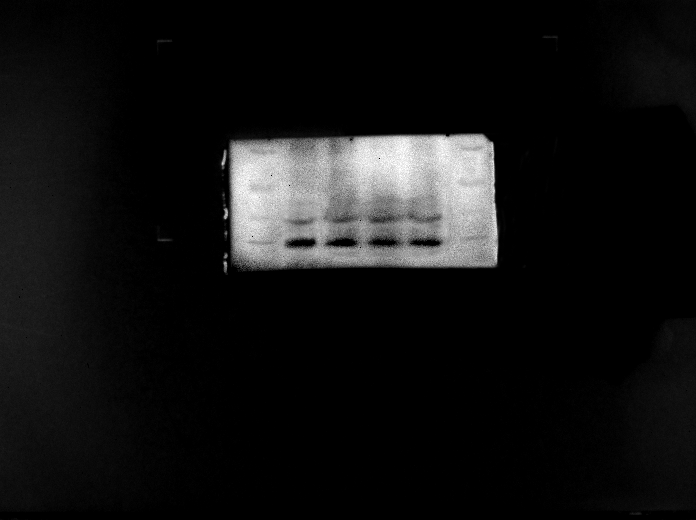

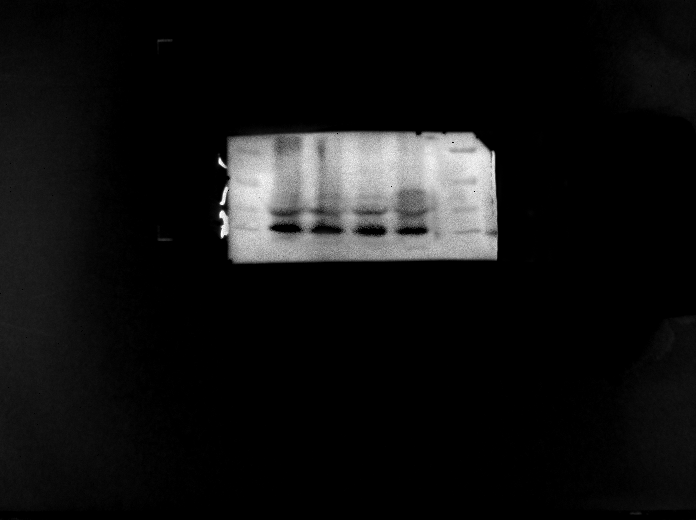

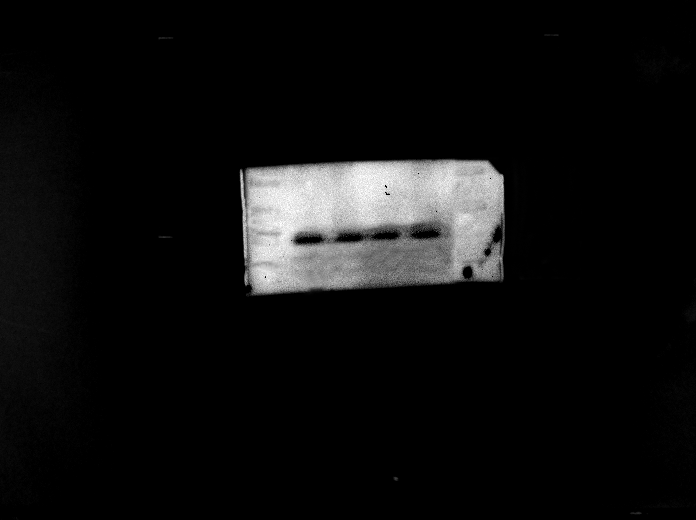


Kidney p-c-jun


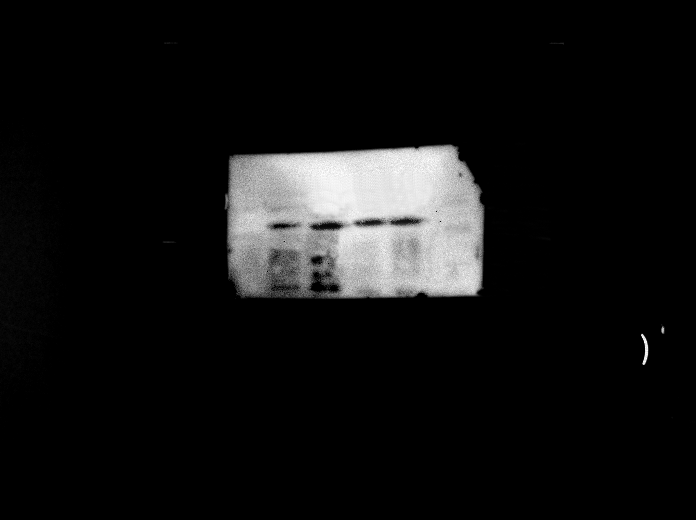

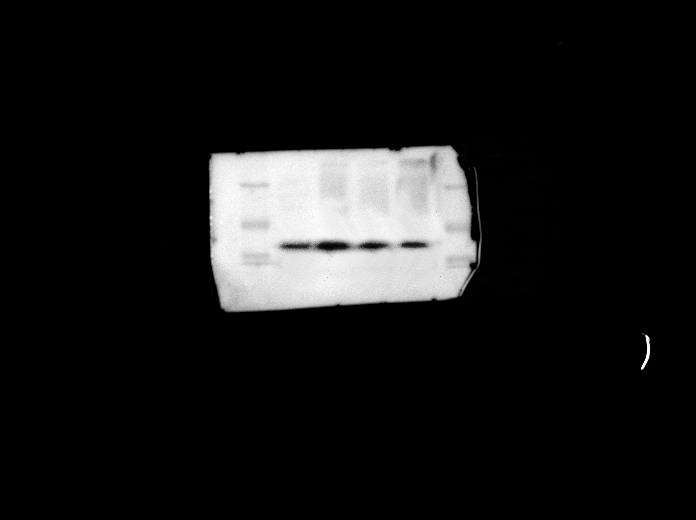

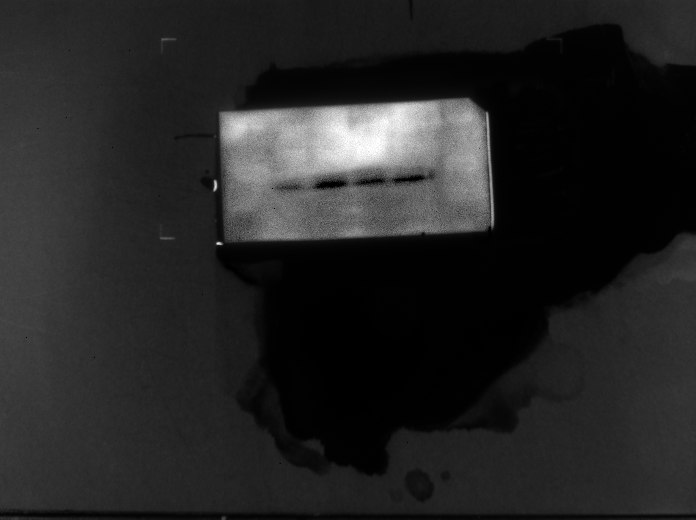


Kidney GAPDH


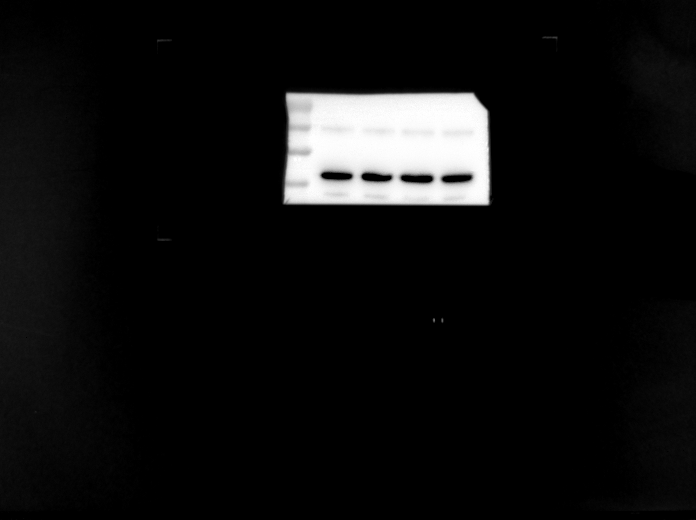

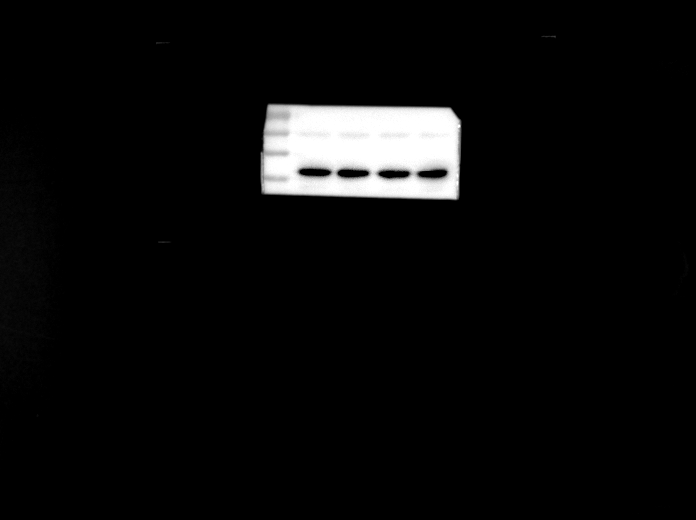

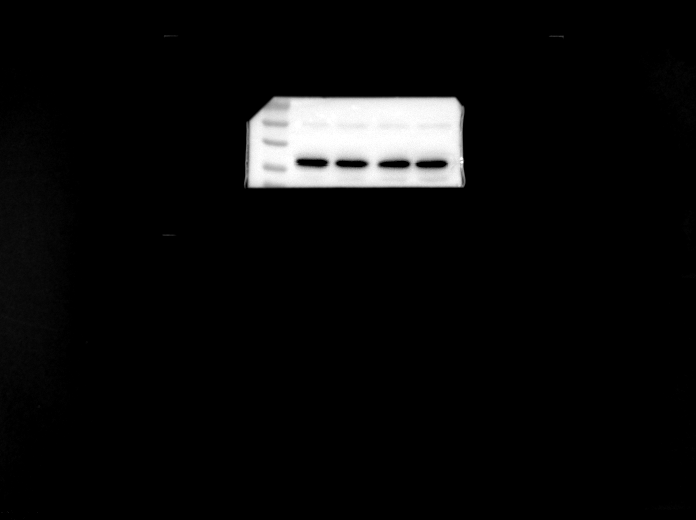

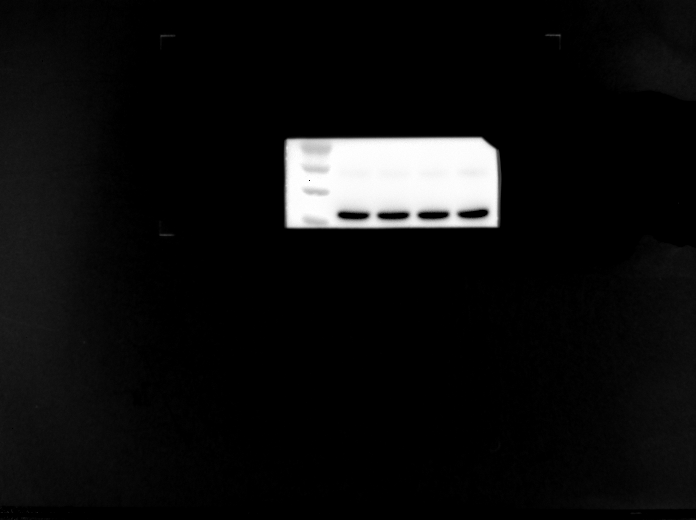

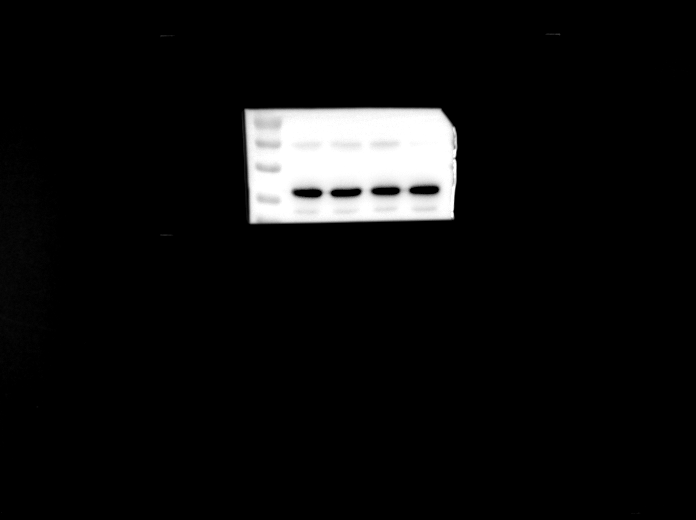

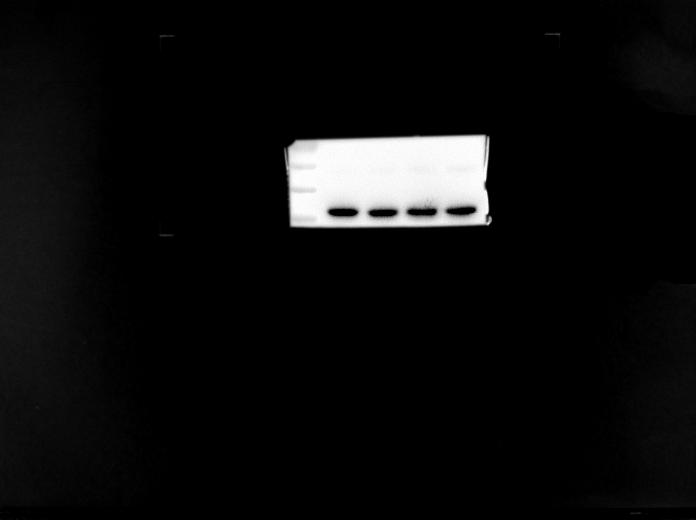

Supplement: Supplementary file 3 [file Table1.DOCX]
